# Vision sculpts a continuum of L2/3 cell types in the visual cortex during the critical period

**DOI:** 10.1101/2023.12.18.572244

**Published:** 2023-12-18

**Authors:** Fangming Xie, Saumya Jain, Salwan Butrus, Karthik Shekhar, S. Lawrence Zipursky

**Affiliations:** 1 Department of Biological Chemistry, Howard Hughes Medical Institute, David Geffen School of Medicine, University of California, Los Angeles, Los Angeles, CA 90095, USA; 2 Department of Chemical and Biomolecular Engineering, Helen Wills Neuroscience Institute, California Institute for Quantitative Biosciences (QB3), University of California, Berkeley, Berkeley, CA 94720, USA; 3 Faculty Scientist, Biological Systems and Engineering Division, Lawrence Berkeley National Laboratory, Berkeley, CA 94720, USA; 4 These authors contributed equally; 5 Lead contact

## Abstract

We previously reported that vision specifies Layer 2/3 (L2/3) glutamatergic cell-type identity in the primary visual cortex (V1). Using unsupervised clustering of single-nucleus RNA-sequencing data, we identified molecularly distinct L2/3 cell types in normal-reared (NR) and dark-reared (DR) mice, but the two sets exhibited poor correspondence. Here, we show that classification of cell types was confounded in DR by vision-dependent gene programs that are orthogonal to gene programs underlying cell-type identity. A focused clustering analysis successfully matches cell types between DR and NR, suggesting that cell identity-defining gene programs persist under vision deprivation but are overshadowed by vision-dependent transcriptomic variation. Using multi-tasking theory we show that L2/3 cell types form a continuum between three cell-archetypes. Visual deprivation markedly shifts this distribution along the continuum. Thus, dark-rearing markedly influences cell states thereby masking cell-type-identities and changes the distribution of L2/3 types along a transcriptomic continuum.

## Introduction

**Figure 1.**
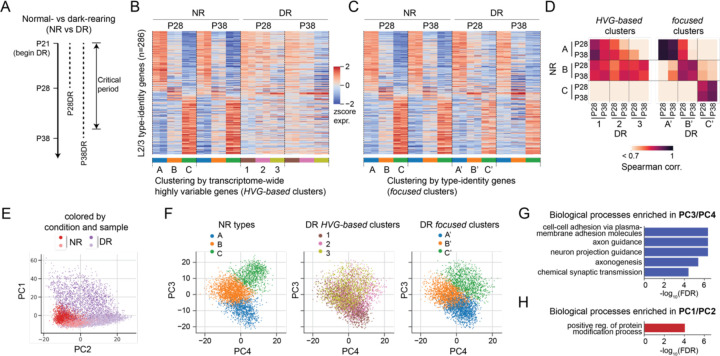
Dark-rearing-induced transcriptomic variations orthogonal to L2/3 cell-type-specific programs masked cell-type identities. **A.** Normal- and dark-rearing (NR and DR) paradigm employed in Cheng et al., 2022^8^. **B-C.** Gene expression profiles (n=286 identity genes; Table S1) of L2/3 glutamatergic neuron types under NR and DR. Clustering in DR was performed based on transcriptome-wide highly variable genes (*HVG-based* clusters; panel **B**) or the signatures of L2/3 identity genes found in NR (*focused* clusters; panel **C**). **D**. Pairwise Spearman’s correlation coefficients between NR and DR types based on L2/3 identity genes. **E-F.** PCA embeddings of P28 NR and DR transcriptomes colored by condition and sample (**E**) and by types **(F)**. PC1 and PC2 separate conditions and samples, while PC3 and PC4 separate based on L2/3 type identity. **G-H.** Gene ontology (GO) analysis showing biological processes enriched in top 100 PC3 and PC4 genes (**G**) and in top 100 PC1 and PC2 genes (**H**). GO terms with FDR < 1e-4 are shown.

Early sensory experiences influence the development of neural circuitry throughout the mammalian brain^1–3^. In the primary visual cortex (V1), visual experience is required to establish the circuitry for binocular vision^4,5^. This vision-dependent development of binocular circuits occurs in mice after eye-opening and is most prominent between postnatal days (P) 21 and 35, a developmental window known as the critical period^4,6,7^. Classically, the influence of early visual experience has not been examined at the level of the many cell types that form the building blocks of V1 circuitry. To address this gap, we previously performed single-nucleus RNA-seq (snRNA-seq) in normal- and dark-reared (NR, DR) mice at key time points spanning the critical period (Figure 1A)^8^. Using computational methods, we reconstructed the postnatal maturation of V1 cell types in NR mice and compared these profiles with DR mice. This enabled us to identify the cell types and gene expression programs that were impacted by vision. For the majority of cell types, DR had little impact on molecular identity. However, DR had a pronounced effect on glutamatergic cell types in the supragranular layers (L) 2/3/4. L2/3 glutamatergic neurons could be grouped into three clusters (A, B and C), which occupied three adjacent sublayers within L2/3 (upper, middle and lower, respectively). In DR mice, however, we found three different transcriptomic clusters (1, 2 and 3) with little correspondence to types A, B and C in NR. These transcriptomic changes were mirrored by altered spatial organization of type-specific gene markers and functional defects, suggesting that visual deprivation during the critical period selectively disrupted L2/3 cell type identities.

To gain deeper understanding of the lack of correspondence between L2/3 cell types in NR and DR, we revisited our published dataset using different computational approaches. Our results reaffirm importance of vision for L2/3 maturation, while clarifying the nature of vision-deprivation induced changes in genes and cell types. We show that DR markedly alters L2/3 transcriptomes, but in gene expression space, the predominant vision-dependent changes are along an axis that is orthogonal to L2/3 cell type-specific signatures. In an unbiased clustering, this orthogonal program masked cell-type identities. A focused analysis, however, shows that cell identity signatures are preserved in DR and are masked by vision-dependent gene programs that dominate the transcriptomic variance. Importantly, the recovery of consistent cell types in both conditions provided a foundation to precisely investigate the cell type-specific effects of vision deprivation. First, visual deprivation differentially impacted the transcriptomes of types B and C more than type A, and blurred the distinctions between types A and B. Second, we detect a higher frequency of type A cells at the expense of type B cells in DR. These vision-dependent effects can be clearly understood using a framework that treats L2/3 cells as a transcriptomic continuum rather than discrete cell types. By invoking a recently proposed framework known as multi-tasking theory^9^, we show that L2/3 cells represent a genuine continuum bounded by three “archetypes” (A, B and C), with visual deprivation altering the distribution of L2/3 cells along this continuum. Taken together, these results highlight the role of sensory experience during the critical period in shaping the continuous variation within a subclass of cortical neurons.

## Results

### Identification of L2/3 cell types in DR mice

In sc/snRNA-seq analysis, cell types are determined by clustering cells based on their expression patterns of highly variable genes (HVGs)^10–12^. By independently clustering snRNA-seq data from NR and DR mice, we previously reported that adult V1 L2/3 glutamatergic neurons comprise three types. We labeled these types A, B and C in NR and 1, 2 and 3 in DR. In each case, these groupings were supported by the expression of hundreds of type-specific genes. Intriguingly, however, the three DR types showed little transcriptomic correspondence with the three NR types.

Types A, B and C in NR mice were distinguished by 286 genes (Table S1) whose expression patterns were consistent at the three time points following the onset of the critical period (P21, P28 and P38) (Figure 1B, Figure S1A). We term these L2/3 type-identity genes. However, these identity genes were not expressed in a type-specific fashion across types 1, 2 and 3 in DR mice (Figure 1B, Figure S1A). This lack of correspondence was seen among L2/3 glutamatergic neuron types, and to a lower extent, among L4 glutamatergic neurons^8^. However, this was not the case for deep layer glutamatergic (L5/6) types, GABAergic types, and non-neuronal types, wherein types mapped 1:1 between NR and DR^8^. Furthermore, these changes were partially reversible during the critical period. In mice that were exposed to 8 hours of light following one week of dark rearing (dark-light, DL), L2/3/4 transcriptomic clusters mapped well to NR types.

To understand the lack of correspondence of L2/3 types between NR and DR, we hypothesized that rather than completely abolishing type-specific programs, DR induces transcriptomic changes that mask type-specific signals. To assess this possibility, we performed a “focused” clustering of L2/3 neurons using only the identity genes (n=286; Table S1). As a control, applying this procedure in NR and DL mice yielded clusters that mapped well to the HVG-based clusters reported previously^8^ (Figure S1B,D; 90% similarity in NR and 78% similarity in DL). In DR, however, focused clusters diverged from HVG-based clusters (Figure S1C; <50% agreement). Unlike the HVG-based clusters, the focused clusters in DR mapped 1:1 with the NR types (A, B, C), and retained NR type-specific signatures (Spearman correlation = 0.91; Figure 1C-D, Figure S1E). To reflect this correspondence, we named the three focused clusters in DR as A’, B’ and C’. Together, this suggests that the co-expression patterns of L2/3 identity genes found in NR are preserved in DR.

### DR-induced transcriptomic variation masked L2/3 cell type identities

To characterize the full set of gene expression programs that underlie transcriptomic variation among L2/3 glutamatergic neurons in NR and DR, we performed Principal Component Analysis (PCA) using a set of HVGs (n=6,360) (Methods). We focused on the top four principal components (PCs), which exhibited a clear spectral gap from the rest of the modes (Figure S2A). PC1 and PC2 separated cells based on sample and rearing condition (Figure 1E). DR cells exhibited greater variation than NR cells along PC1 and PC2, which also separated HVG-based clusters 1, 2 and 3, rather than focused types A, B, and C (Figure S2B-C). By contrast, PC3 and PC4 separated cells based on focused cluster identity in both NR and DR (Figure 1F). This suggests that despite not being the dominant mode of variation, type-specific signals are highly consistent between NR and DR.

Gene ontology analysis showed that PC3 and PC4 were enriched for genes associated with cell adhesion, axon guidance, and chemical synaptic transmission (Figure 1G; Figure S2D), which may contribute to differences in neuronal cell-type identity. By contrast, genes driving PC1 and PC2 were enriched in protein modification and dephosphorylation (Figure 1H, Figure S2E) as well as immediate-early genes^13^, which may reflect shifts in cell state associated with experience-induced changes in activity (Figure S2G). Consistent with gene ontology, we found PC1 and PC2 genes have less than 1% overlap (1/200 genes in common; odds ratio = 0.1, p=0.002; Fisher exact test using HVGs as background) with the type-identity genes, whereas PC3 and PC4 genes have >55% overlap (114/200 genes in common; odds ratio = 46, p<10^−10^) (Figure S2F). In summary, PC1 and PC2 represent cell state differences, and PC3 and PC4 capture differences in cell type identity.

The PCA results provide insight into the discordance of HVG-based clusters between NR and DR in L2/3. For NR, HVGs overlapped largely with genes driving PC3 and PC4, while HVGs for DR overlapped with genes driving PC1 and PC2. The focused clustering emphasized genes driving PC3 and PC4, enabling the identification of consistent types between the two conditions. The discordance in HVG-based clustering is specific to L2/3 glutamatergic neurons, as this procedure identifies consistent cell types between NR and DR for all other subclasses, as noted in our prior results^8^.

### DR differentially impacted L2/3 cell types and blurred distinctions between them

**Figure 2.**
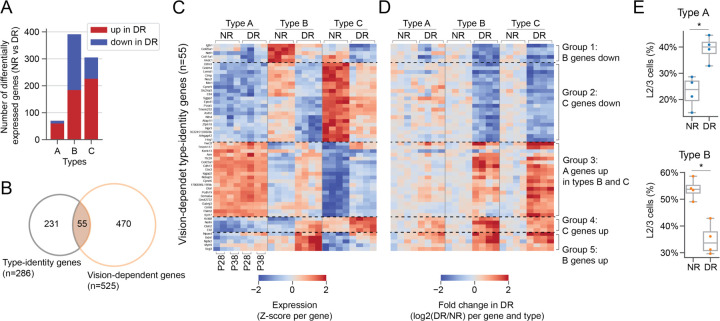
Dark-rearing differentially impacted L2/3 types and blurred the distinction between them. **A.** Number of vision-dependent genes in each L2/3 type. **B.** Overlap between vision-dependent genes and identity genes. **C-D.** Expression profiles of genes that were vision-dependent and in Table S1. Expression was quantified as the z-scored expression level per gene across all samples (**C**), and as log2 fold change with respect to the mean expression level in NR (**D**). **E.** The relative abundance of type A cells (top panel) and type B cells (bottom panel) among L2/3 glutamatergic neurons in NR and DR. * represents p<0.05.

We next examined visual deprivation-induced changes in gene expression. A differential gene expression analysis between NR and DR within matched types (A vs A’; B vs B’; C vs C’) identified 70 vision-dependent genes in type A, 391 genes in type B, and 305 genes in type C (Fold change >2 and FDR <0.05; Figure 2A; Figure S3A-B). Types B and C displayed about seven-fold more vision-dependent genes than type A even when each type was downsampled to contain the same number of cells (Figure S3C). Among vision-dependent genes, 10% (55/525) overlapped with the identity genes (Table S1) and among identity genes, 20% (55/286) were vision-dependent (Figure 2B). The 55 identity genes that were vision-dependent fell into five groups (Figure 2C-D). Two groups corresponded to B- (n=5; Group 1) and C-specific (n=21; Group 2) genes whose expression levels were down-regulated in DR. Two groups contained B- (n=5; Group 5) and C-specific (n=4; Group 4) genes up-regulated in DR. Unlike B- and C-specific genes, A-specific genes were only present in in one group (n=20, Group 3), which were up-regulated in type B cells, but did not change in type-A cells. Overall, type-A cells were largely unaffected by DR (Figure S3D), type C cells lost some of their specific features, and type-B cells acquired A-like features accompanied by a change in B-like features.

These changes at the gene expression level were accompanied by a marked shift in the proportion of cells assigned to each of the three types between NR and DR. The proportion of type-B cells decreased from ~50% in NR to ~35% in DR, while type-A cell abundance increased from ~25% to ~40% (Figure 2E; Figure S3E). The proportion of type C was relatively unaffected. Together, these results indicate that DR differentially impacted L2/3 types B and C, and blurred the distinctions between types A and B.

### L2/3 cell types are organized along a transcriptomic continuum bounded by three archetypes

**Figure 3.**
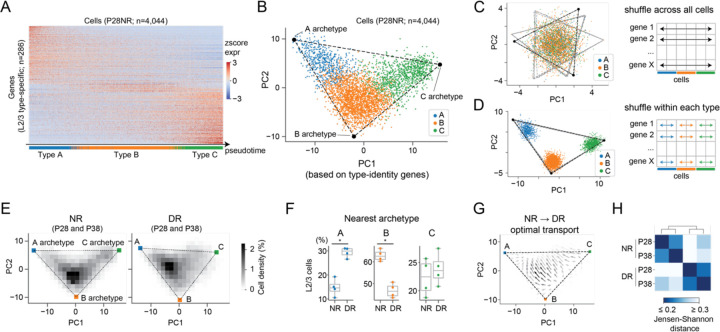
Dark-rearing shifted cells along the transcriptomic continuum of L2/3 cell types. **A.** Expression profiles of L2/3 type-specific genes (n=286) across all L2/3 glutamatergic neurons (n=4,044; P28NR). Expression was quantified as z-scored, log- and size-normalized counts from scRNA-seq. Cells were ranked by diffusion pseudotime, and colored by type identity (annotation bar, bottom). **B.** PCA embedding (PC1 and PC2 based on identity genes) of L2/3 glutamatergic neurons (n=4,044; P28NR). Cells are colored by type. The triangular bound were inferred using the published framework^9^ (see Methods). **C-D.** Same as (B) after shuffling each gene independently across all cells (**C**), and after shuffling each gene independently across cells within each type (**D**), respectively. The gray lines represent triangular fits using 80% of cells, randomly downsampled 10 times independently. **E.** Cell density within PC1 and PC2 space for NR (left) and DR (right). **F.** Boxplot showing the proportion of cells nearest to each archetype under NR and DR. **G.** Optimal transport from the NR to DR distribution. **H.** Pairwise Jensen-Shannon distances between the type frequency distribution among samples.

While the grouping into types A, B and C is a convenient framework for NR mice, it may not fully capture the changes occurring under DR conditions. We previously reported that L2/3 cell types form a continuum characterized by the graded expression of hundreds of genes^8^. This continuum organization is also evident when L2/3 cells are ordered by diffusion pseudotime (DPT)^14^ calculated based on the 286 identity genes (Figure 3A; Figure S4). We hypothesized that treating L2/3 cells as a continuum, rather than discrete types, may provide clearer insight into the effects of visual deprivation.

First, to gain confidence that L2/3 cells are indeed continuously organized, we applied multi-tasking theory^9^, a framework to analyze transcriptomic continuums (Methods). According to this theory, a continuous distribution of cells in the space of genes are bounded by a polygon. The vertices of the polygon represent archetypes, and cells that form the continuum are mixtures of the archetypes. Indeed, L2/3 neurons form a continuum bounded by a triangle, whose vertices represent the A, B, and C archetypes (Figure 3B). Shuffling gene expression values across all cells, irrespective of type, no longer fits a triangle and all cells collapse towards the center (p<0.001; t-ratio test) (Figure 3C, Figure S5A,B,D).

It is well known that snRNA-seq data is affected by multiple sources of noise. We developed a computational procedure to distinguish between two scenarios: a genuine continuum vs. discrete types that are seemingly continuous due to noise in the data. In this procedure, we compute PCA embeddings after shuffling expression values independently for each gene within each cluster (Figure 3D; Figure S6A-D). We first tested this approach via simulation. When applied to clusters that span a continuum, the shuffling procedure splits continuums into discrete clusters as long as noise levels in the data are low to intermediate (Figure S6E). In contrast, when the clusters are already discrete it has no effect regardless of noise level (Figure S6F). Therefore, if L2/3 cells were comprised of discrete clusters that appeared continuous because of noise, this procedure should have no effect. However, in contrast to this, and consistent with the continuum scenario, we found that applying this shuffling procedure to our data splits L2/3 cells into three distinct clusters (Figure 3D; Figure S5C). This result supports our view that L2/3 cells form a genuine transcriptomic continuum rather representing discrete types that appear continuous because of noisy data.

### DR redistributed B-like cells to A-like cells

That L2/3 cells form continuous rather than discrete types clarifies how vision regulates their transcriptomic organization. L2/3 transcriptomes from both NR and DR mice occupied similar triangular regions bound by the same three archetypes in PC space. Individual cells, however, distributed differently between conditions with a shift away from the archetype B towards A in DR (Figure 3E-F). These trends were robust across biological replicates (Figure S7A-C). An optimal transport analysis^15,16^ from NR to DR predicted a redistribution of cells from archetype B towards A, and less movement for cells already located near archetypes A and C (Figure 3G). Moreover, we found that the distributions of cells at P28 and P38 NR were more closely related to each other than they were to the distributions at P28 and P38 DR (Figure 3H). Finally, the distribution of cells in DL mice were similar to that seen in NR (Figure S7D-E). Taken together, these results indicate that the transcriptional programs defining types A, B and C cells are plastic, such that cells can “move” to proximal regions in the transcriptomic continuum in a vision-dependent manner.

In summary, dark-rearing disrupted L2/3 glutamatergic cell types along a transcriptomic continuum. It caused a major redistribution of cell type-specific gene expression programs, such that cells moved away from archetypical B-like programs towards A-like programs. This is consistent with the results that type-A identity genes were up-regulated in type-B cells in DR.

## Discussion

We previously showed that L2/3 glutamatergic cells can be categorized into three transcriptomic cell-types (types A, B and C). Surprisingly, dark-reared cell-types (types 1, 2 and 3) whose gene expression signatures substantially differed from types A, B and C. Here, we reanalyzed our transcriptomic data to look deeper into the effect of vision on gene expression and to define relationships between types identified under DR and NR conditions.

Analyses presented in this study reaffirm the vision-dependent development of L2/3 cell types in the primary visual cortex while clarifying how visual deprivation alters transcriptional programs. We found that gene programs underlying cell type-identity and those activated by dark rearing are largely orthogonal. The deprivation-induced state changes were large enough to mask the distinctions between L2/3 types. A focused clustering that only considered the 286 “identity” genes defined in NR successfully identified corresponding types in DR. We leveraged this correspondence to delineate the influence of vision on the L2/3 cell type continuum. Thus, in some circumstances a focused approach may reveal important features of gene expression not picked up by unsupervised methods.

The transcriptional diversity of L2/3 cells is a continuum, and partitioning this continuum into three discrete types is a crude approximation. Using multi-tasking theory, we showed that the distribution of L2/3 cells in gene expression space was bounded by a triangle with vertices representing archetypes A, B and C. L2/3 cells span the continuum in between these archetypes. Dark-rearing redistributes cells within the continuum, which shifts cells away from the B-archetype, closer to A. This change is consistent with our previously reported results from smFISH experiments, which showed that dark-rearing leads to an extended domain of A-type marker (*Cdh13*) expression, accompanied by a reduction in cells positive for a B-type marker (*Trpc6*). In addition, DR leads to a decrease in the expression of C-type genes, without shifting the identity of C-like cells specifically towards A or B. Taken together, these results support the idea that vision is required to maintain the proper distribution of cells in the continuum bounded by archetypes A, B and C.

How a transcriptomic continuum is created among L2/3 glutamatergic neurons in V1 over development remains an outstanding question. Here, we show that vision is required to maintain the proper arrangement of cells along the continuum. A more detailed analysis of gene expression programs and signaling pathways over development in the presence and absence of normal vision will be key to identifying the order of molecular events that give rise to the observed continuum in V1 neurons.

The presence of a continuum, which is sculpted by vision raises an important question: what role does a continuum play in the proper function of V1? Multitasking Theory, developed in the context of ecology and tissue biology of liver and intestines^9,17^, suggests that transcriptomic continuums aid the division of labor among cells in a tissue responsible for multiple functions. For continuums bound by a polygon, cells close to each vertex (archetype) are specialized for a particular function, while cells in the middle are “generalists”, that can perform multiple functions at the cost of being sub-optimal for any single function. When gradients in gene expression space are also correlated with position, this can create spatial domains within a tissue, each of which is suited for a unique complement of functions. Consistent with this idea, transcriptomic continuums have recently been found to be a common trait in the mammalian brain, and they are often correlated with spatial and physiological continuums^18–25^. In particular, the physiological and morphological features of L2/3 neurons in binocular V1 vary as a continuum along the pial-ventricular axis^26^. It has also been shown that L2/3 neurons in V1 project their axons to neighboring higher visual areas in a complex non-random fashion^27^. Together, these findings suggest that vision controls the function and connectivity of L2/3 glutamatergic neurons in V1 by conferring upon them a continuum of cell type identities. As L2/3 neuron types have selectively expanded in the primate cortex, continuums of cell types may be particularly well suited to change by experience and in this way contribute to the complexity of cortical circuitry.

## Methods

### DR sample preparation

Although no new mouse experiments were performed in this study, we briefly describe the protocol for dark rearing mice that was employed previously^8^to generate the datasets analyzed here. Mice were dark reared starting post-natal day 21 (P21), which marks the beginning of the ocular dominance critical period. Prior to P21, mice were reared in cages in a room with a 12-hour light/dark cycle. At P21, mouse cages were moved to a separate room and placed inside a dark-box lined on the inside and the outside with a black rubberized fabric with edges sealed by tape and fabric to avoid any light entry. Mice were dark reared in this setup for 7 days (P28DR group) or 14 days (P38DR group). During this period, any handling of the cages was performed in the dark with room lights off, door crevices sealed from exterior light, and in dim red light, which is invisible to the mice. At the end of dark-rearing, mouse cages were taken out of the dark-box, wrapped in black-rubberized fabric, and immediately transported to an adjacent room for euthanization. Mice were placed inside a plexiglass chamber with isoflurane gas for anesthetization, followed by euthanasia. During this period mice were exposed to ambient light for a maximum of 2 minutes.

### snRNA-seq data acquisition and normalization

Cell-by-gene count matrices from the previous study were downloaded from Gene Expression Omnibus (GEO) with the accession number GSE190940^8^. Cell type labels for HVG-based clusters were downloaded from the associated Github repository https://github.com/shekharlab/mouseVC. Raw count matrices were normalized as described before. Transcript counts within each cell were rescaled to sum up to 10,000. A pseudo-count of 1 was added to the normalized transcript counts for each gene within each cell, followed by log10-transformation. For PCA and clustering, log10-transformed counts were z-scored across cells for each gene. Both NR and DR samples were processed in the same way, and we did not apply any of the commonly used batch integration techniques (e.g. ^28,29^) in our analyses.

We also reproduced the differential expression analysis from the previous study to identify L2/3 identity genes (n=286) listed in Table S1. Briefly, the expression of each gene was compared in one type versus others. We used the function *tl.rank_genes_groups* from the Scanpy package with the following criteria to identify a gene as DE: a) FDR < 0.05 with Wilcoxon rank-sum test; b) fold increase > 2 in one type compared with the two other types combined, and c) the gene was expressed in > 30% cells in the up-regulated type.

### Selection of highly variable genes (HVGs)

The raw count matrices have n=53,801 genes to start with, including both protein-coding and non-coding genes. We considered only the n=21,222 genes that had non-zero counts in more at least 10 L2/3 glutamatergic neurons in P28 mice, combing both NR and DR samples. Of these, we selected a subset as highly variable genes based on the following procedure:

- For each gene, we computed the ratio of the variance and the mean of the normalized expression counts. Under a Poisson distribution, variance-mean ratio is expected to be one. Indeed, the variance-mean ratio is overall stable across orders of magnitude difference in mean expressions.
- To select highly variable genes with different baseline (mean) expressions, we grouped genes into decile-bins according to mean expression level, and for each bin we selected the top 30% genes with the most variance-mean ratio. 6,360 genes were selected, and most (270/286) L2/3 type-specific genes were included.

### Focused clustering

We used K-means clustering with the python package *sklearn* and the function *sklearn.cluster.Kmeans* to cluster L2/3 glutamatergic neurons, using the z-scored expression values of L2/3 type-specific genes (n=286; Table S1) as features. We applied K-means clustering with K=3 on each sample separately, including P28NR, P28DR, P28DL, P38NR, and P38DR. For each sample, the resulting three clusters were readily matched with types A, B, C in P28NR based on the Spearman correlation coefficient across genes (*scipy.stats.spearmanr*). Therefore, the three clusters in each sample were independently named as types A, B, C (focused types).

### Principal component analysis (PCA)

PCA was perfomed on each condition separately (P28NR, P28DR, and so on) using z-scored HVGs as features using python package *sklearn* and its function *sklearn.decomposition.PCA*. In addition, we applied PCA combining both P28NR and P28DR samples together. We evaluated the similarity between two PCA eigenvectors $v_{i}$ and $v_{j}$ as the absolute value of their dot product $\left(\left|v_{i}\cdot v_{j}\right|\right)$. As the eigenvectors are orthonormal, this value is bounded between 0 and 1.

To characterize the L2/3 continuum via multi-tasking theory and changes due to visual deprivaion (Figure 3), we applied PCA combining all conditions (P28NR, P28DR, P28DL, P38NR, P38DR) using z-scored L2/3 type-specific genes (n=286; Table S1) as features. Focusing on L2/3 type-specific genes and combining all conditions allowed us to study the L2/3 continuum in a consistent representation across all conditions.

### Gene ontology (GO) analysis

We used the web-based tool *EnrichR*: https://maayanlab.cloud/Enrichr/ for GO analysis. All enrichment analyses were performed by comparing a gene list of interest with the default background gene set. Top 10 enriched GO terms of Biological Process that were statistically significant with adjusted p-value < 0.05 were shown.

### Identification of differentially expressed genes (DEGs) between NR and DR

In scRNA-seq analysis, it is common to treat individual cells as samples in statistical tests for identifying DEGs. Often, however, this approach leads to many statistically significant genes as even modest effect sizes can appear unlikely (low p-values) under the “no effect” null hypothesis owing large cell numbers. Therefore, as a conservative measure to compare changes between NR and DR, we chose to regard the biological samples, rather than single cells, as independent data points.

We compared NR vs DR across independent biological samples, with each condition having 4 independent biological samples: P28 rep1, P28 rep2, P38 rep1 and P38 rep2. Raw counts from cells of the same types and samples were aggregated to produce pseudo-bulk profiles. We only considered genes with mean expression (in counts per million transcripts; CPM) > 10 in at least one type in either NR or DR. We performed independent sample t-test (*scipy.stats.ttest_ind*) on the pseudo-bulk profiles between NR and DR for each subclass. The resulting p-values were adjusted by the Benjamini-Hochberg procedure to calculate the false discovery rate (FDR). Effect sizes were quantified as the log2 fold change (in CPM) in DR compared with NR. DEGs were defined as those with FDR<0.05 and $|{\text{log}}_{2}FC|>1$.

### Pseudotime analysis

We computed a diffusion embedding of L2/3 cells to understand their continuous organization. We first computed the top 50 PCs using z-scored L2/3 identity genes, and used these reduced dimensional coordinates to generate a k-nearest-neighbor (k=50) graph between cells. The graph was built using the function *Scanpy.pp.neighbors*. Next, we generated a diffusion map^14^ using *Scanpy.tl.diffmap* with default parameters. We assigned the cell with the smallest PC1 as the root cell, and then ran diffusion pseudotime using *Scanpy.dpt* with default parameters. As a result, each cell was assigned a pseudo-temporal coordinate, and cells were ranked by the increasing order of pseudotime.

Next, we then ranked L2/3 type-identity genes according to their expression along the pseudotime coordinates. We defined the pseudotime of a gene $\left(T_{g}\right)$ as the weighted average of cell pseudotime,

$$T_{g}=\sum_{c}w_{gc}t_{c},$$

where $t_{c}$ is the pseudotime of the cell $\left(c\right)$, and $w_{gc}$ is the weight of the cell 𝑐 contributing to the gene g. The weight of a gene sums to one and is defined by expression level,

$$w_{gc}=\frac{x_{gc^{\prime }}}{\sum_{c^{\prime }}x_{gc^{\prime }}}.$$

where $x_{gc}$ is the size and log normalized expression. Gene pseudotime is defined at P28NR and is kept fixed across different time points and conditions to give a consistent representation for comparisons between conditions.

### Analysis of transcriptomic gradients using multi-tasking theory

We used the python package *py_pcha*: https://github.com/ulfaslak/py_pcha to fit a triangular boundary to L2/3 transcriptomes in PC1 and PC2 space that was reduced from n=286 L2/3 type-specific genes. The algorithm infers a triangular boundary by fitting a principal convex hull from the data. We chose parameters *delta=0* and *noc=3*. The same procedure was applied to each condition separately and to shuffled data.

We evaluated the significance of the triangular fit by t-ratio test as proposed by the multi-tasking theory^9^. T-ratio is the ratio between the area of the convex hull of the data and that of the principal convex hull (the triangular bound in our case). We calculated the area of the convex hull using the python package *scipy* and its function *scipy.spatial.ConvexHull*. We tested the significance of the observed t-ratio by comparing it to t-ratios of shuffled data by permuting each gene independently across all cells. P-value was computed based on 1000 shuffles.

### Simulations of gradients and discrete types

First, we describe our procedure to simulate transcriptomic continuums. Let cells and genes be ranked, such cells with a particular ranking express genes with a similar ranking. Let i be an integer that represents the cell ranking, which goes from 1 to $N_{c}$, with $N_{c}$ denoting the total number of cells. Let j be an integer that represents the gene ranking, which goes from 1 to $N_{g}$, with $N_{g}$ denoting the total number of genes. We then use $p_{i}=\frac{i}{N_{C}}$ and $p_{j}=\frac{j}{N_{g}}$ to represent the normalized rankings such that both the gene rankings and cell rankings go from 0 to 1. We consider a model where the expected expression level of gene j in cell i is a Gaussian such that:

$$\lambda_{ij}=e^{-{\left(\frac{p_{i}-p_{j}}{\varepsilon }\right)}^{2}},$$

where ε denotes the level of noise. Gene counts are then drawn from a scaled Poisson distribution parametrized by $\lambda_{ij}$:

$$c_{ij}\sim s\cdot Poisson\left(\lambda_{ij}\right).$$

For our simulation, we chose $N_{g}=60$, $N_{c}=600$, s=100, with different ε values between 0.1 and 1. Random numbers were generated using the python package *numpy.random*.

Second, for simulating discrete types, we assumed a model wherein each type is distinguished by a set of “marker” genes. Let i be a cell and $C\left(i\right)$ be the cell type it belongs to. Let j be a gene and $C\left(j\right)$ be the cell type of which it is a marker. The expected expression level of gene j in cell i was modeled by a binary matrix:

$$\lambda_{ij}=\left\{\begin{array}{ll} 1 & if\;C\left(i\right)=C\left(j\right)\\ \varepsilon & if\;C\left(i\right)\neq C\left(j\right) \end{array}\right.,$$

where ε denotes the amount of leaky expression, i.e., the level of noise. ε takes values between 0 and 1. The larger ε is, the noisier the types are. The actual count matrix was drawn from a scaled Poisson distribution parametrized by $\lambda_{ij}$,

$$c_{ij}\sim s\cdot Poisson\left(\lambda_{ij}\right).$$

We simulated three discrete types (A, B, C), each with 20 marker genes and containing 200 cells, with different ε values between 0.1 and 1. To assign an order between types, among the 20 marker genes, 6 were shared between neighboring types, such that 6 type-A markers were also expressed in type B, and 6 type-B markers were also expressed in type C, and so on.

### Quantification of cell redistribution in DR

To visualize the cell density distribution in PC1 and PC2 space, we used the python package *seaborn.histplot*. We confirmed that the distributions were robust with respect to a range of bin-widths, and chose bin-widths of 1~1.5 for visualization. We used the Jensen-Shannon (JS) divergence, a symmetric metric to characterize the difference between two probability distributions, to measure the level of similarity between cell-density distributions within the L2/3 continuum in different rearing conditions and time points. This metric was calculated using the python package *scipy* and its function *scipy.spatial.distance.jensenshannon*.

Optimal transport analysis was performed using the python package *POT*: https://pythonot.github.io/. The program computes the optimal transport map connecting the empirical distributions of NR cells and DR cells in PC1 and PC2 space^16,30^. The inputs were cell coordinates in PC1 and PC2 space for NR and DR respectively. We first computed pairwise distance matrix between NR and DR cells using *ot.dist* following default parameters. We then used *ot.emd* to calculate the transport map. The result is a matrix whose elements denote transport couplings between each pair of NR and DR cells, which is a proxy for their transcriptomic correspondence. We visualized this result by a coarse-grained vector field as follows. First, PC1 and PC2 space were meshed into 15-by-15 bins. Each 2D bin was represented by an arrow, where the length of the arrow represents the mean moving directions and magnitudes of all local cells (vector mean), and the darkness of the arrow represents the relative number of cells it represents.

### Data and code availability

- All data was downloaded from GEO with the accession number GSE190940.
- Code to reproduce L2/3 type specific genes (Table S1, n=286) was obtained from the GitHub repository: https://github.com/shekharlab/mouseVC
- Code to reproduce the figures in the manuscript was deposited in the following GitHub repository: https://github.com/FangmingXie/vision_and_visctx

## Supplementary Material

Supplement 1

Supplement 2
