# Supplementary material for: Spatial profiling of the interplay between cell type- and vision-dependent transcriptomic programs in the visual cortex
Source: bioRxiv. 2024 Oct 17:2023.12.18.572244. Originally published 2023 Dec 18. Preprint. [Version 2] doi: 10.1101/2023.12.18.572244 (PMC10769288; doi:10.1101/2023.12.18.572244)
Supplement: Supplement 6 [file NIHPP2023.12.18.572244v2-supplement-6.pdf]

bioRxiv preprint doi: https://doi.org/10.1101/2023.12.18.572244; this version posted October 17, 2024. The copyright holder for this preprint (which was not certified by peer review) is the author/funder, who has granted bioRxiv a license to display the preprint in perpetuity. It is made available under aCC-BY 4.0 International license.

Supplementary Figures

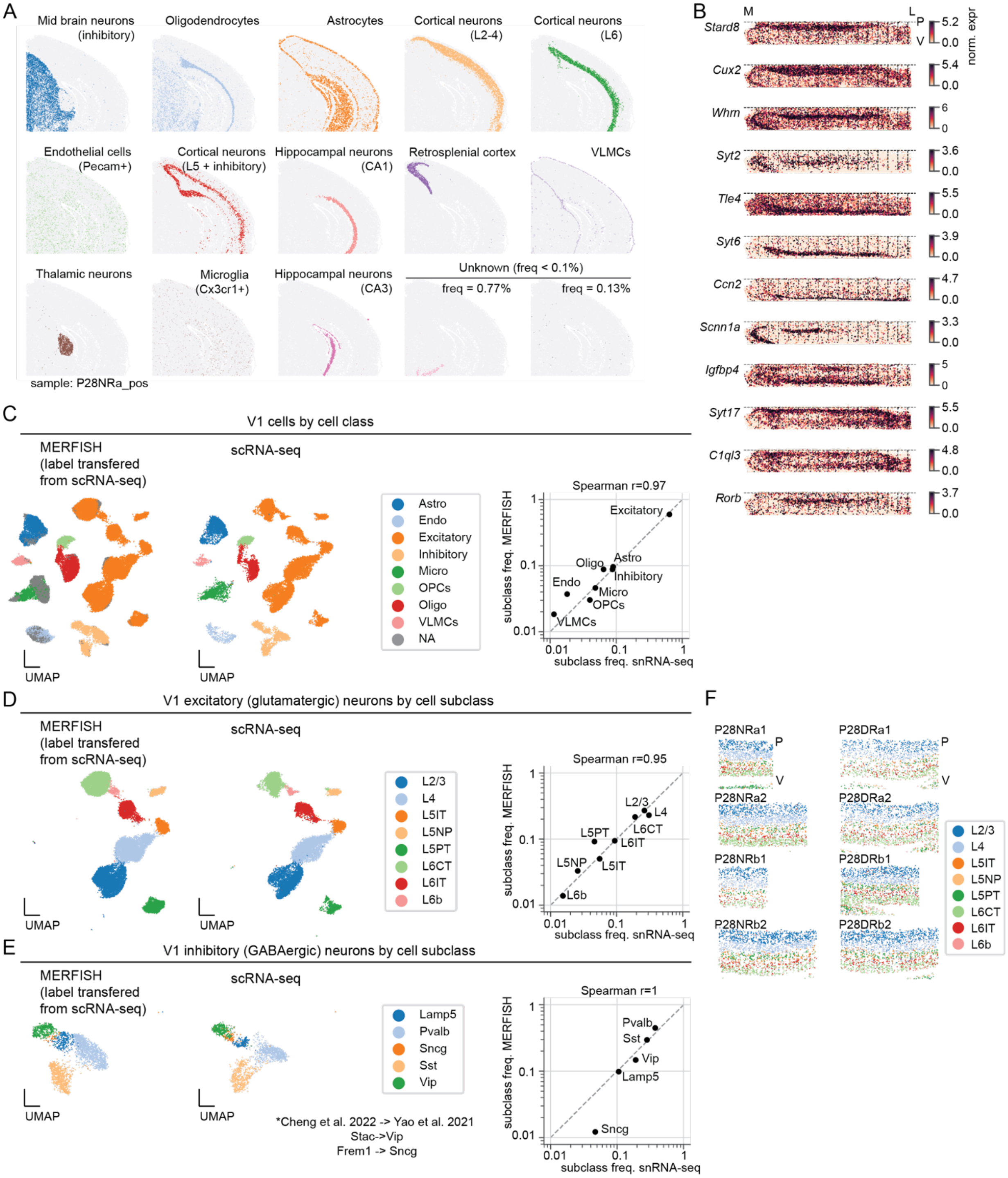

Figure S1. Related to Figure 1. MERFISH identifies major anatomical structures of the brain and cell subclasses in V1.

(A) Spatial distributions of individual cell clusters identified based on gene expressions and spatial proximity (Related to **Figure 1B**). (B) *In situ* gene expression patterns across the

bioRxiv preprint doi: https://doi.org/10.1101/2023.12.18.572244; this version posted October 17, 2024. The copyright holder for this preprint (which was not certified by peer review) is the author/funder, who has granted bioRxiv a license to display the preprint in perpetuity. It is made available under aCC-BY 4.0 International license.

straightened cortex. (C) V1 cell classes represented in UMAP obtained from integrating MERFISH (left panel; this study) and snRNA-seq (middle panel (9)) using Harmony (14). Right panel compares cell type proportions between snRNA-seq and MERFISH. 19.6% of MERFISH cells could not be classified (marked as NA and colored gray) due to their low quality and were filtered from downstream analyses. These cells are likely low-quality glia as they are co-clustered with non-neuronal cells. (D-E) Subclasses of V1 glutamatergic neurons (D) and GABAergic neurons (E) represented in UMAP embeddings obtained from integrating MERFISH (left panel; this study) and snRNA-seq (middle panel (9)) using Harmony (14). Right panels compare cell type proportions between snRNA-seq and MERFISH. (F) Spatial distributions of V1 glutamatergic subclasses.

bioRxiv preprint doi: https://doi.org/10.1101/2023.12.18.572244; this version posted October 17, 2024. The copyright holder for this preprint (which was not certified by peer review) is the author/funder, who has granted bioRxiv a license to display the preprint in perpetuity. It is made available under aCC-BY 4.0 International license.

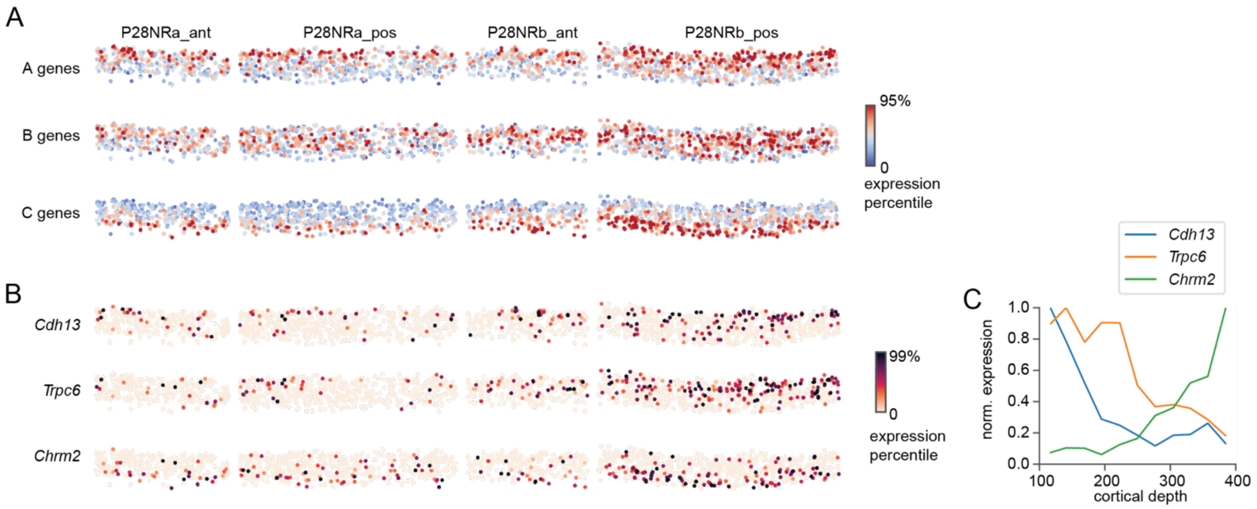

Figure S2. Related to Figure 1. *In situ* distributions of L2/3 type-identity genes.

(A) *In situ* expression patterns of types A, B and C identity genes. (B) *In situ* expression patterns of *Cdh13*, *Trpc6* and *Chrm2*. *Cdh13* marks type A, *Trpc6* marks type B, and *Chrm2* marks type C. (C) Line plots showing the distribution of marker gene expressions along the cortical depths spanning L2/3.

bioRxiv preprint doi: https://doi.org/10.1101/2023.12.18.572244; this version posted October 17, 2024. The copyright holder for this preprint (which was not certified by peer review) is the author/funder, who has granted bioRxiv a license to display the preprint in perpetuity. It is made available under aCC-BY 4.0 International license.

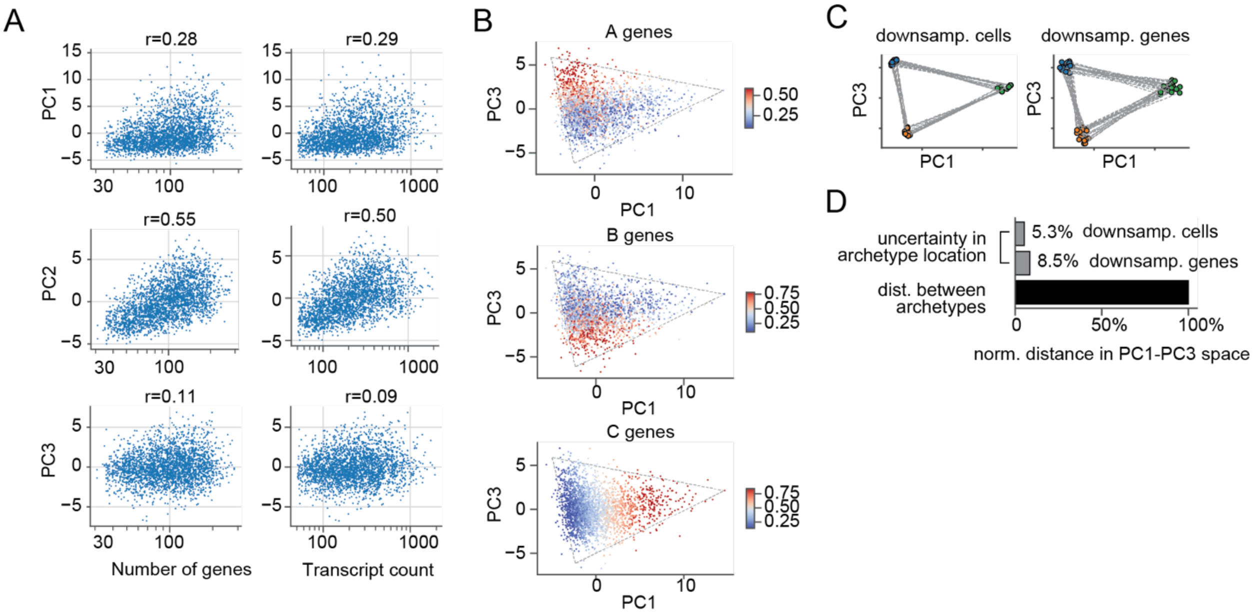

Figure S3. Related to Figure 2. Low dimensional representation of L2/3 cell types in MERFISH and inference of the bounding triangle from MERFISH data alone.

(A) Scatter plots of PC1 (upper), PC2 (middle) and PC3 (lower) vs. the number of detected genes (left) and transcript count (right panel), respectively. PC1 is calculated using the 170 type-identity genes measured by MERFISH. PC2 correlates with these technical variables much more than PC1 and PC3. (B) V1 L2/3 cells embedded in PC1 and PC3. Cells are colored by the mean z-scored expression levels of type-A genes (upper panel), type B genes (middle panel) and type C genes (lower panel), respectively. (C-D) Stability of the bounding triangle. The bound triangle was inferred in several instances involving random down-sampling to 80% of cells (C, left panel) and 80% of genes (C, right panel). The stability of archetype locations are quantified by the % variation in archetype coordinates relative to the typical inter-archetype distances in these trials. Distances are measured by Euclidean distance in PC1-PC3 space. These inferences were done on MERFISH data, recapitulating results from snRNA-seq showed in Figure 2.

bioRxiv preprint doi: https://doi.org/10.1101/2023.12.18.572244; this version posted October 17, 2024. The copyright holder for this preprint (which was not certified by peer review) is the author/funder, who has granted bioRxiv a license to display the preprint in perpetuity. It is made available under aCC-BY 4.0 International license.

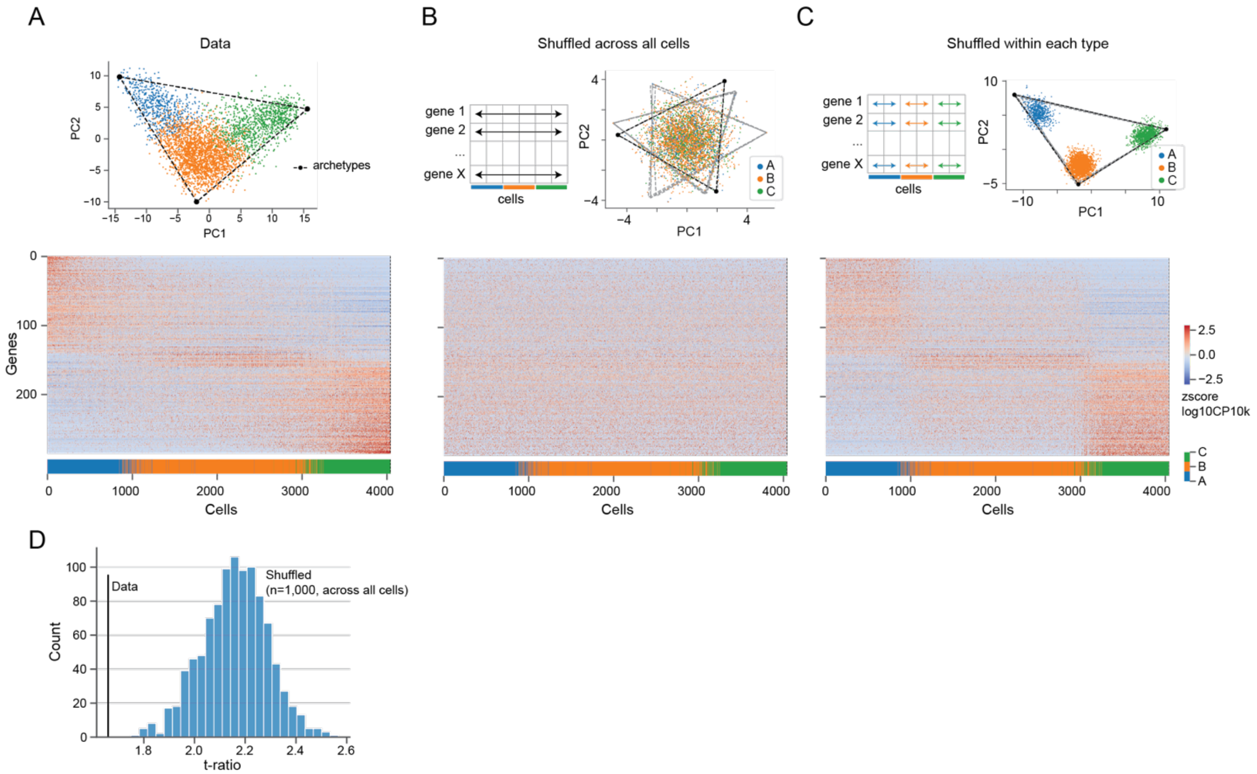

Figure S4. Related to Figure 2. Data shuffling disrupted the geometry of the L2/3 continuous transcriptomic manifold.

(A) *Upper panel:* PCA embedding of L2/3 glutamatergic neurons in snRNA-seq using 286 type-identity genes. Cells were colored by type assignment based on the original clustering (9). The bounding triangle is inferred from archetypal analysis. *Lower panel:* expression profiles of L2/3 type-identity genes across all L2/3 glutamatergic neurons (n=4,044; P28NR). Expressions were quantified as z-scored, log- and size-normalized counts from scRNA-seq. Cells were ranked by diffusion pseudo-time (DPT; (19, 20)), and colored by type assignment. (B) Same as (A) but after shuffling each gene independently across all cells. (C) Same as (A) but after shuffling each gene independently across cells within each type. (D) Histogram of T-ratios for the data and shuffled data (n=1,000; shuffled across all cells). T-ratio is the ratio between the area of the convex hull and that of the principal convex hull (triangular fit).

bioRxiv preprint doi: https://doi.org/10.1101/2023.12.18.572244; this version posted October 17, 2024. The copyright holder for this preprint (which was not certified by peer review) is the author/funder, who has granted bioRxiv a license to display the preprint in perpetuity. It is made available under aCC-BY 4.0 International license.

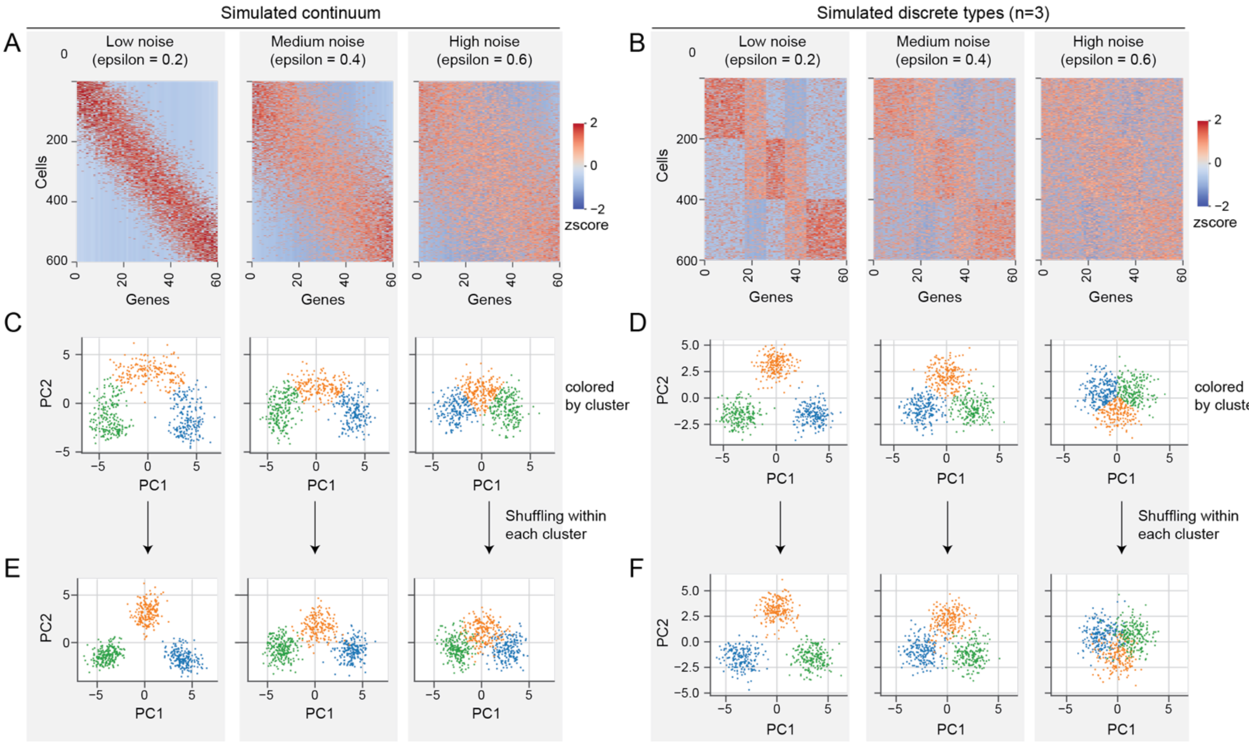

Figure S5. Related to Figure 2. A data shuffling procedure that split a continuum while preserving discrete types.

(A-B) Expression profiles (gene by cell) for simulated continua (A) and simulated discrete types (B) each with varying degrees of noise level (parameterized by epsilon; see **Methods**). Expression was quantified as z-scores. (C-D) PCA embeddings (PC1 and PC2) of simulated continua (C) and simulated discrete types (D). Cells were colored by type. (E-F) Same as (C-D) but after shuffling each gene independently across all cells within each type. Shuffling genes within a cluster splits a continuum into separate clusters when noise is low (epsilon = 0.2 ~ 0.4). The same procedure has no effect on discrete types whatsoever.

bioRxiv preprint doi: https://doi.org/10.1101/2023.12.18.572244; this version posted October 17, 2024. The copyright holder for this preprint (which was not certified by peer review) is the author/funder, who has granted bioRxiv a license to display the preprint in perpetuity. It is made available under aCC-BY 4.0 International license.

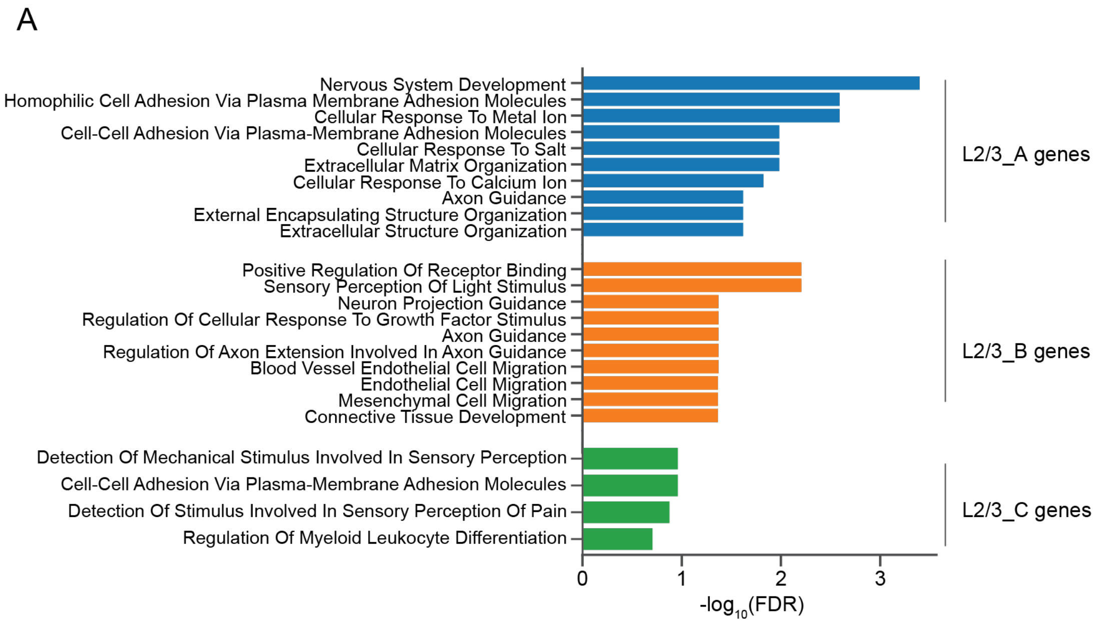

Figure S6. Related to Figure 2. Gene Ontology analysis identify enriched biological processes for type-identity genes.

(A) Enriched biological processes for types A, B and C identity genes, respectively.

bioRxiv preprint doi: https://doi.org/10.1101/2023.12.18.572244; this version posted October 17, 2024. The copyright holder for this preprint (which was not certified by peer review) is the author/funder, who has granted bioRxiv a license to display the preprint in perpetuity. It is made available under aCC-BY 4.0 International license.

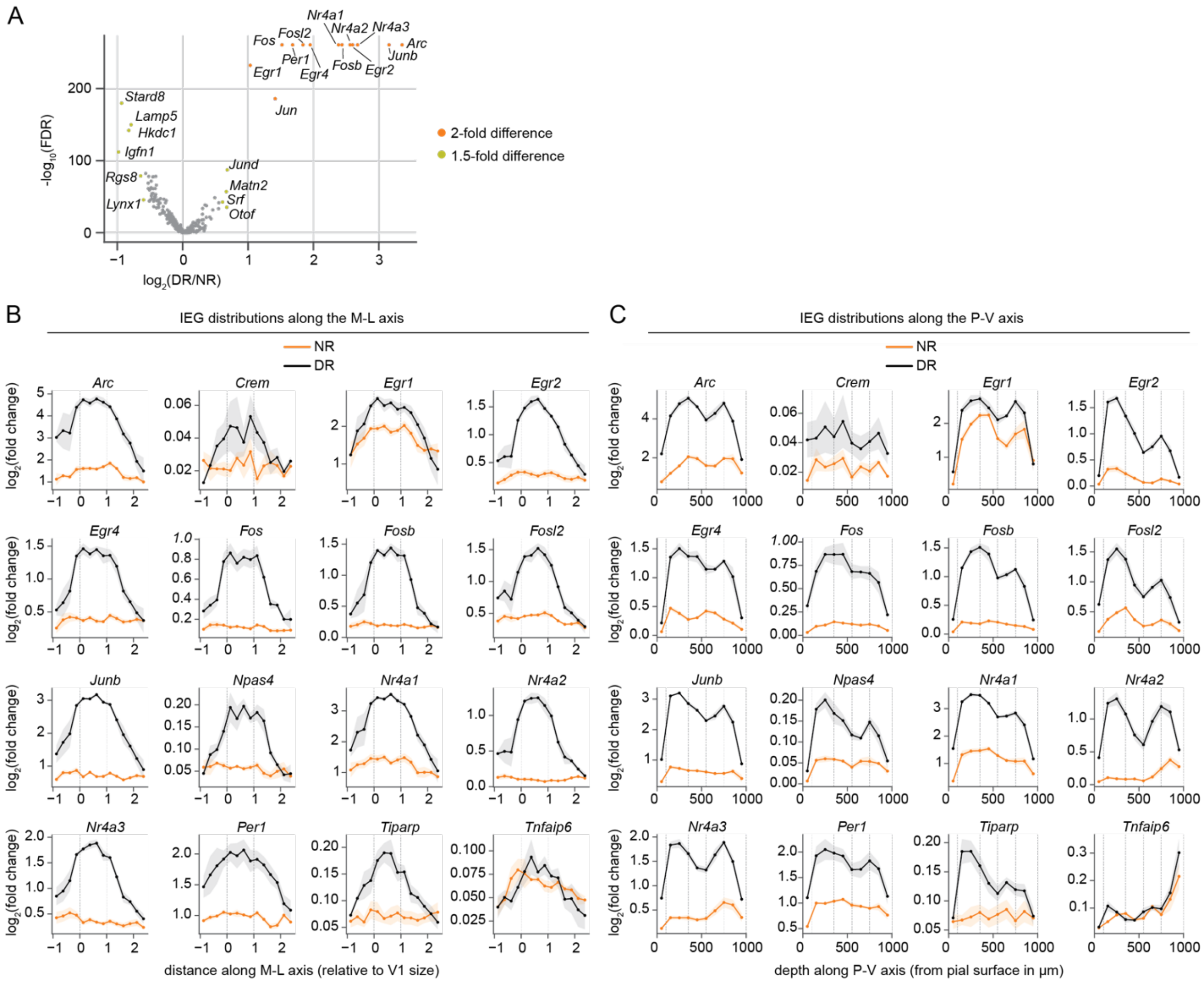

Figure S7. Related to Figure 3. Vision-dependent genes profiled by MERFISH.

(A) Violin plot showing differentially expressed genes between NR and DR L2/3 glutamatergic neurons profiled by MERFISH. (B-C) Mean expression levels of individual IEGs along the M-L axis (B) with V1 in the middle and its flanking regions on the two sides, and along the P-V axis (C) across cortical layers. Lines demarcate V1 (B) and cortical layers (C).

bioRxiv preprint doi: https://doi.org/10.1101/2023.12.18.572244; this version posted October 17, 2024. The copyright holder for this preprint (which was not certified by peer review) is the author/funder, who has granted bioRxiv a license to display the preprint in perpetuity. It is made available under aCC-BY 4.0 International license.

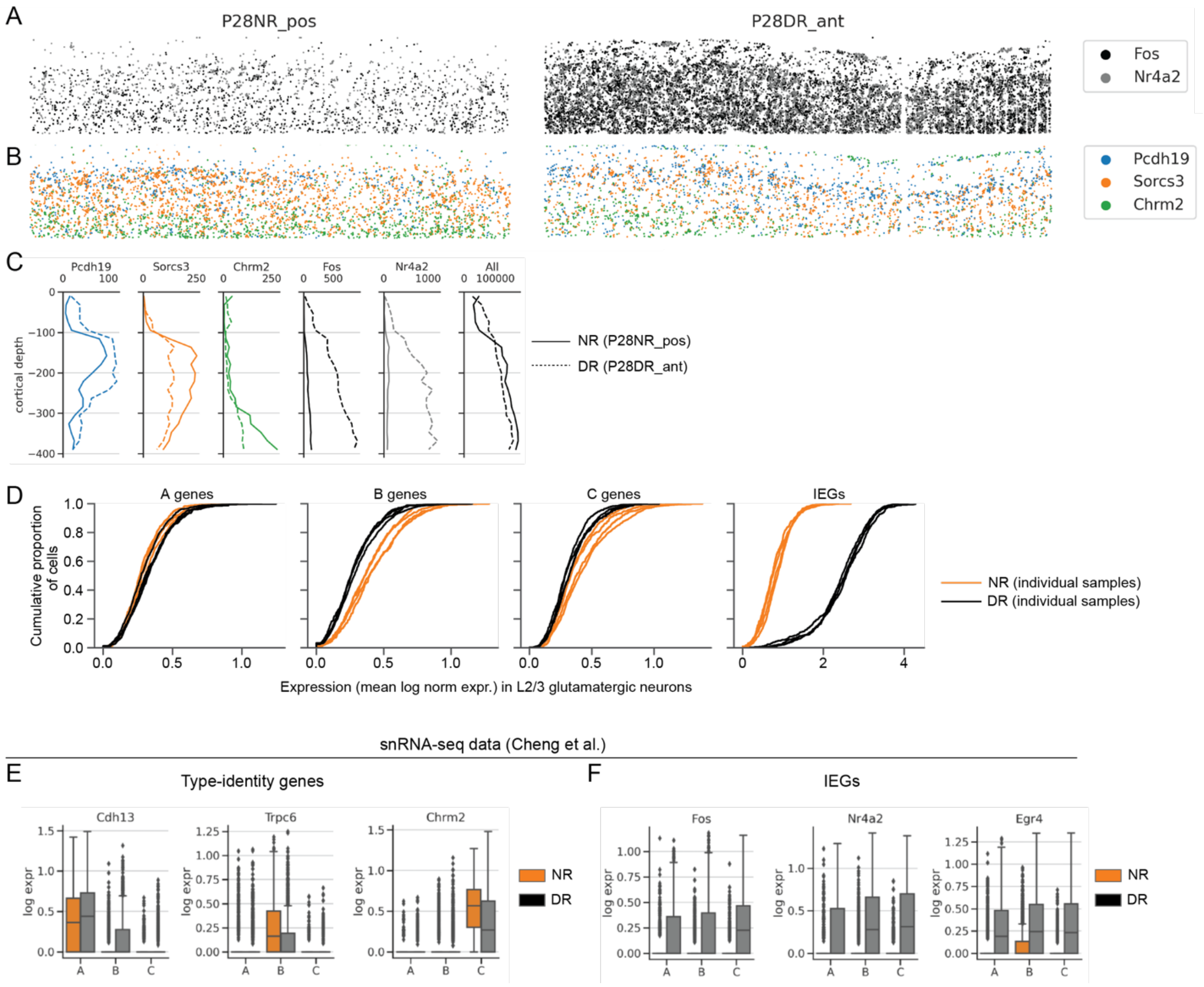

Figure S8. Related to Figure 3. MERFISH detected transcripts in V1 L2/3 for specific genes and gene groups.

(A-B) Detected transcripts of Fos and Nr4a2 (A) and of *Pcdh19*, *Sorcs3*, and *Chrm2* (B) in V1 L2/3 for NR and DR samples. The entire V1 L1-3 area is shown. (C) Quantification of the number of detected transcripts at different cortical depths for genes shown in panels (A-B). (D) Cumulative distribution of gene expression across different gene groups and samples. Samples are colored by NR and DR. Group-level expression is defined as the mean expression level across genes within the group. (E-F) Gene expression distributions (in box plots) of selected type-identity genes (E) and IEGs (F) in L2/3 types from NR and DR mice.

bioRxiv preprint doi: https://doi.org/10.1101/2023.12.18.572244; this version posted October 17, 2024. The copyright holder for this preprint (which was not certified by peer review) is the author/funder, who has granted bioRxiv a license to display the preprint in perpetuity. It is made available under aCC-BY 4.0 International license.

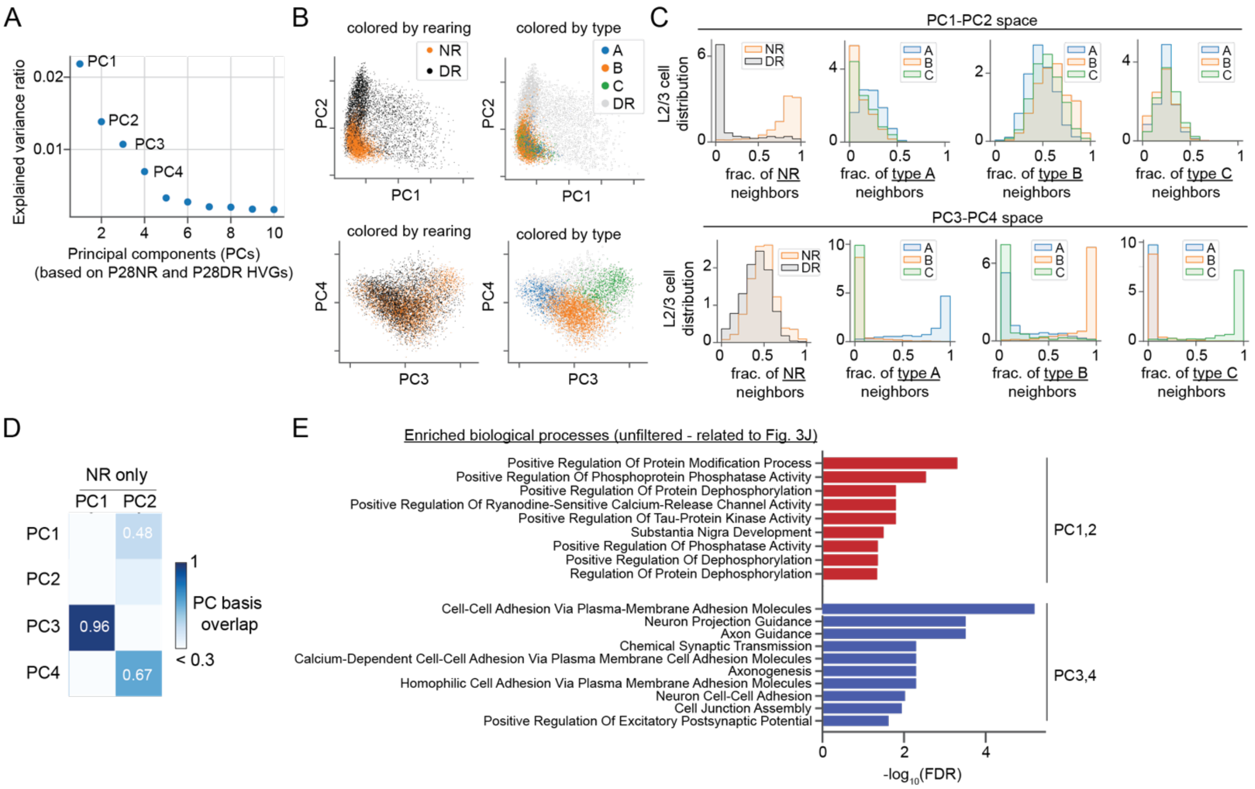

Figure S9. Related to Figure 3. PCA analysis revealed orthogonal components of transcriptomic variations in L2/3 transcriptomes from NR and DR mice.

(A) Fraction of total variance captured within the top 10 principal components (PCs) in NR and DR (P28) L2/3 cells. (B) Distribution of NR and DR cells in PC1-PC2 space (upper) and PC3-PC4 space (lower). PCs are computed from n=6,360 HVGs. Cells are colored by rearing condition (NR vs DR) (left) and by cell type (A, B and C) for NR cells (right). (C) Distribution of neighbor identities. Column 1 shows the fraction of NR neighbors for NR cells (in orange) and DR cells (in black) in PC1-PC2 space (upper panel) and PC3-PC4 space (lower panel). Columns 2-4 show the fraction of types A, B and C neighbors respectively. (D) Pairwise overlap between PCs derived from using both NR and DR cells versus using NR cells only. (E) Enriched biological processes for PC1-PC2 and PC3-PC4 driving genes. This is related to main Figure 3J.

bioRxiv preprint doi: https://doi.org/10.1101/2023.12.18.572244; this version posted October 17, 2024. The copyright holder for this preprint (which was not certified by peer review) is the author/funder, who has granted bioRxiv a license to display the preprint in perpetuity. It is made available under aCC-BY 4.0 International license.

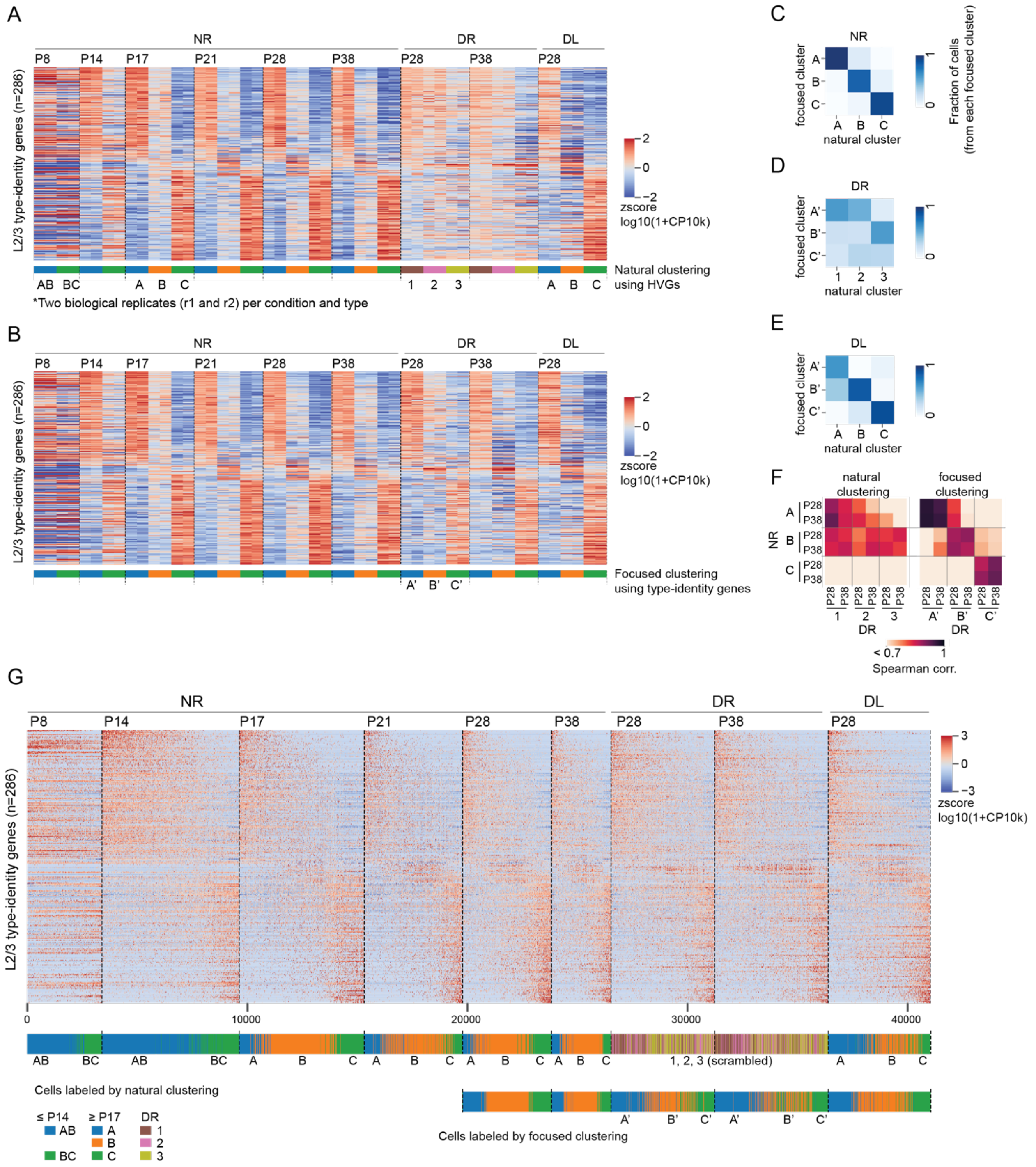

Figure S10. Related to Figure 3. The L2/3 transcriptomic continuum across time and conditions.

(A-B) Expression profiles of L2/3 type-identity genes for clusters based on natural clustering using HVGs (A), and based on focused clustering using type-identity genes (B). Natural clustering using HVGs mask the cell-type signatures in DR, while focused clustering using type-identity genes recover the cell-type signatures. (C-E) Confusion matrix of natural clustering vs focused clustering for NR (C), DR (D) and DL (E), respectively. (F) Pairwise Spearman's

bioRxiv preprint doi: https://doi.org/10.1101/2023.12.18.572244; this version posted October 17, 2024. The copyright holder for this preprint (which was not certified by peer review) is the author/funder, who has granted bioRxiv a license to display the preprint in perpetuity. It is made available under aCC-BY 4.0 International license.

correlation coefficients between NR and DR cell clusters based on L2/3 identity genes. Focused clustering has 1:1 correspondence between NR and DR clusters, while natural clustering does not. (G) Expression profiles of L2/3 type-specific genes for individual cells. Top: Gene expression profiles. Bottom: cells colored by HVG-based and focused clustering respectively. Cells were ordered by diffusion pseudo-time for each condition.

bioRxiv preprint doi: https://doi.org/10.1101/2023.12.18.572244; this version posted October 17, 2024. The copyright holder for this preprint (which was not certified by peer review) is the author/funder, who has granted bioRxiv a license to display the preprint in perpetuity. It is made available under aCC-BY 4.0 International license.

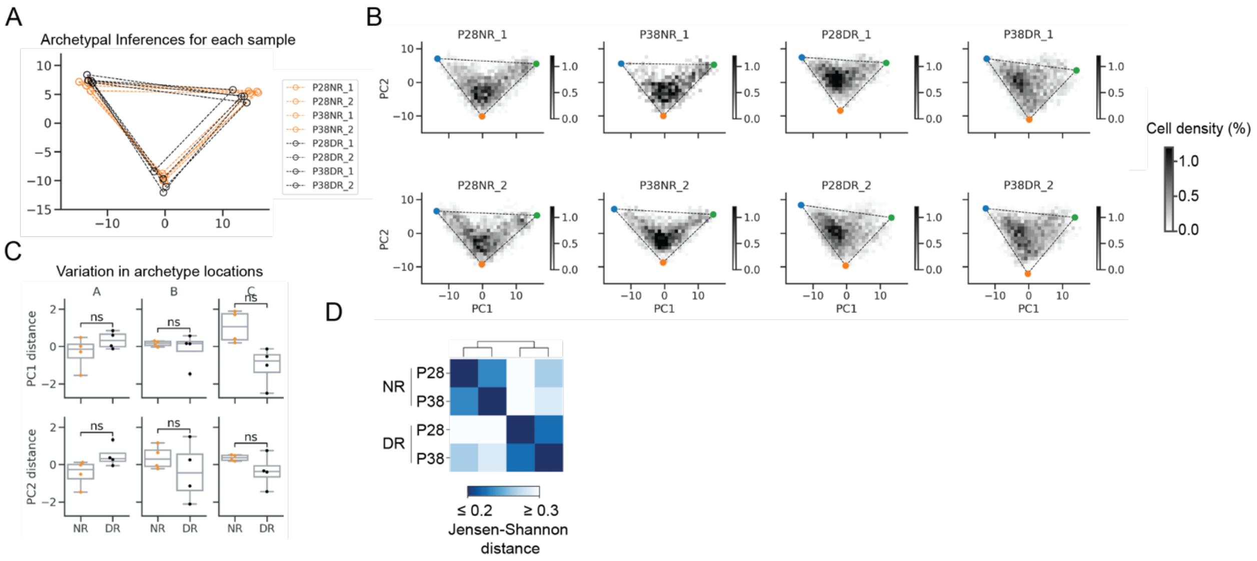

Figure S11. Related to Figure 4. Dark-rearing shifted cells along the continuous transcriptomic manifold of L2/3 cell types (snRNA-seq data).

(A) Inferred L2/3 transcriptomic triangle for individual biological samples. (B) L2/3 cell density plot in PCA embeddings (PC1 and PC2 based on type-identity genes). L2/3 cells from all samples are embedded into the same PC1-PC2 space using type-identity genes. Cell density and the archetypal inference are done separately for each sample. (C) Boxplots showing the variations of archetype locations in PC1-PC2 space. (D) Pairwise Jensen-Shannon distances between samples of different rearing conditions.

bioRxiv preprint doi: https://doi.org/10.1101/2023.12.18.572244; this version posted October 17, 2024. The copyright holder for this preprint (which was not certified by peer review) is the author/funder, who has granted bioRxiv a license to display the preprint in perpetuity. It is made available under aCC-BY 4.0 International license.

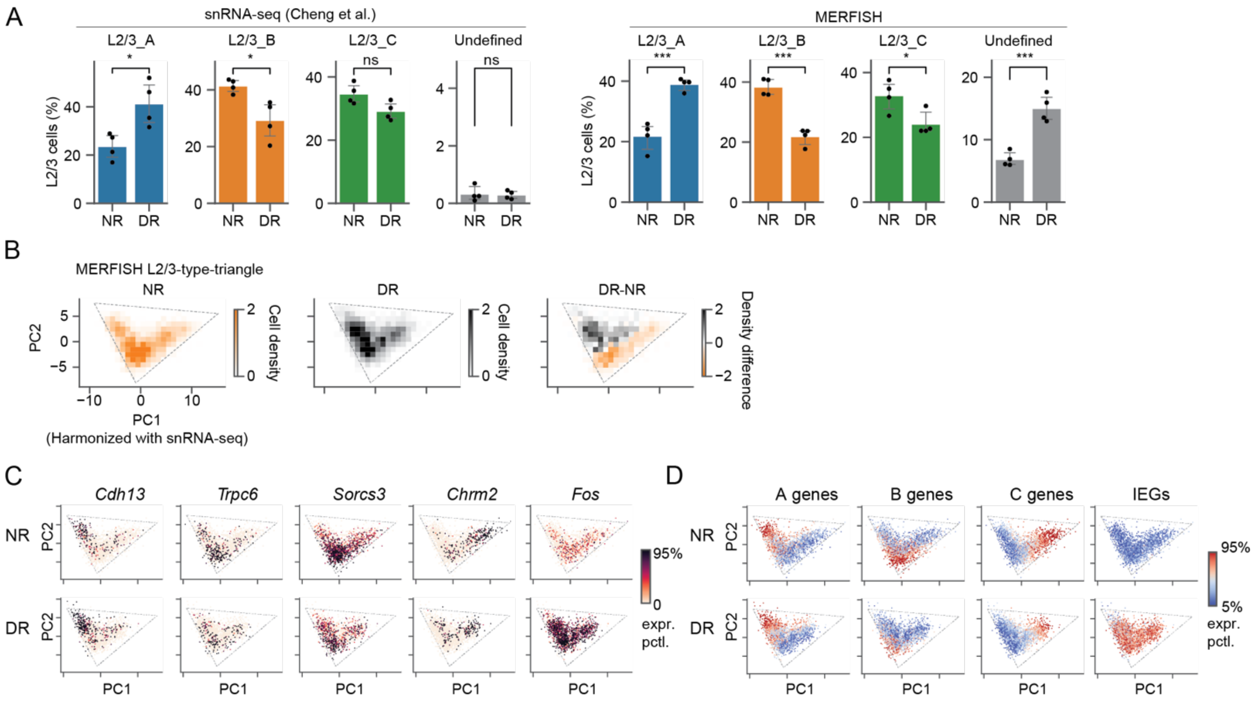

Figure S12. Related to Figure 4. Dark-rearing shifted cells along the transcriptomic manifold of L2/3 cell types (MERFISH data).

(A) Proportion of L2/3 cell types from NR and DR samples profiled by snRNA-seq (left panel) and MERFISH (right panel). Each dot represents a biological sample. Cell type labels are based on the expression levels of type-identity genes (see Methods). (B) Distribution of L2/3 cells within its bounding triangle for NR and DR mice profiled by MERFISH. (C-D) Gene expression patterns for individual genes (C) and groups of genes (D) on the L2/3 type-triangle.

bioRxiv preprint doi: https://doi.org/10.1101/2023.12.18.572244; this version posted October 17, 2024. The copyright holder for this preprint (which was not certified by peer review) is the author/funder, who has granted bioRxiv a license to display the preprint in perpetuity. It is made available under aCC-BY 4.0 International license.

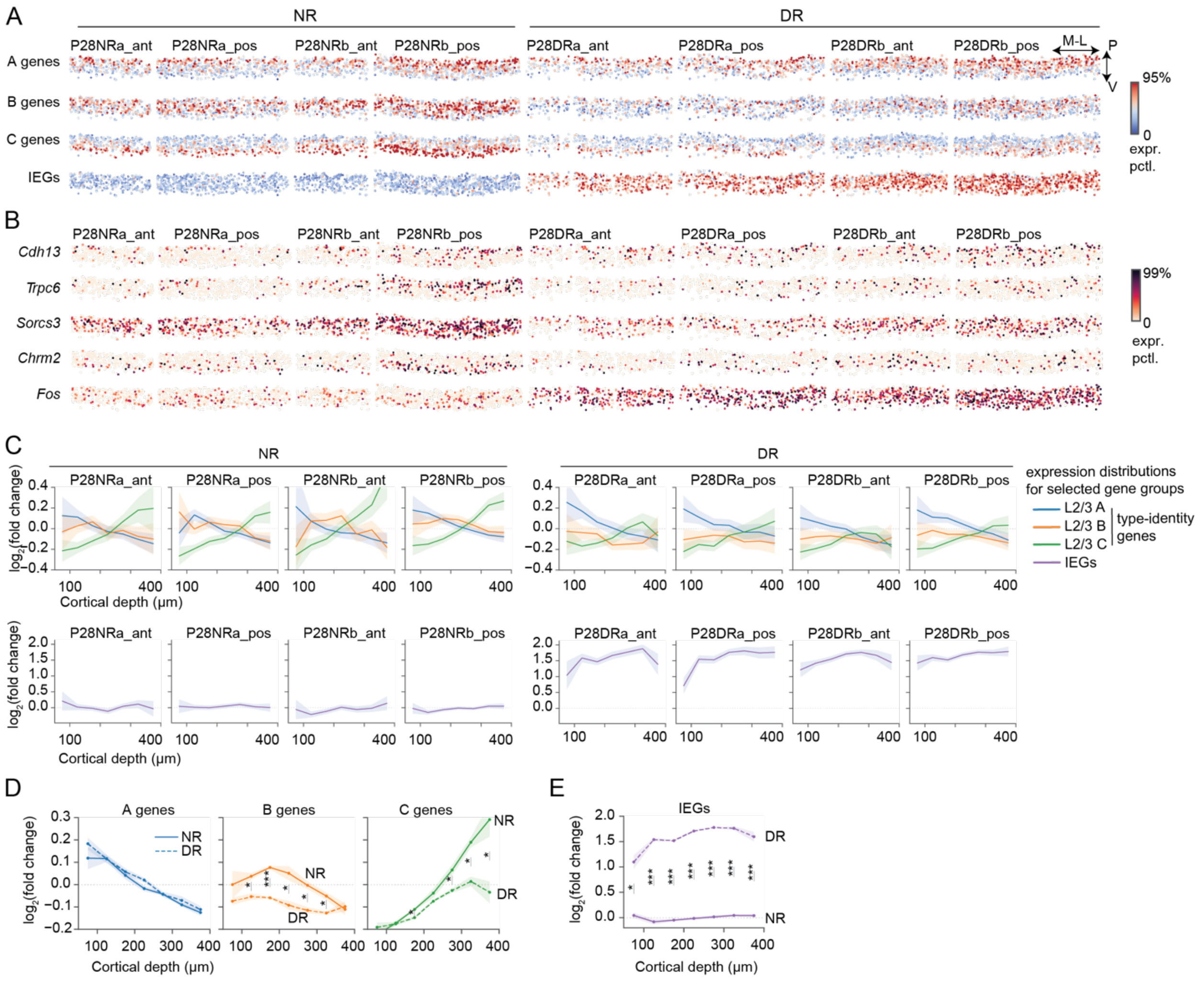

Figure S13. Related to Figure 4. Characterization of *in situ* gene expression in V1 L2/3.

(A-B) in situ expression patterns of different gene groups (A) and individual genes (B) in V1 L2/3. Each row shows one gene (or gene group) and each column one sample. (C) Line plots showing the mean expression levels of type-identity genes (upper panel) and IEGs (lower panel) along the cortical depth for individual samples. (D-E) Line plots comparing the expression levels of type-identity genes (D) and IEGs (E) in NR and DR across cortical depths spanning L2/3. FDR < 0.05 (*) and FDR < 0.001 (***).

bioRxiv preprint doi: https://doi.org/10.1101/2023.12.18.572244; this version posted October 17, 2024. The copyright holder for this preprint (which was not certified by peer review) is the author/funder, who has granted bioRxiv a license to display the preprint in perpetuity. It is made available under aCC-BY 4.0 International license.

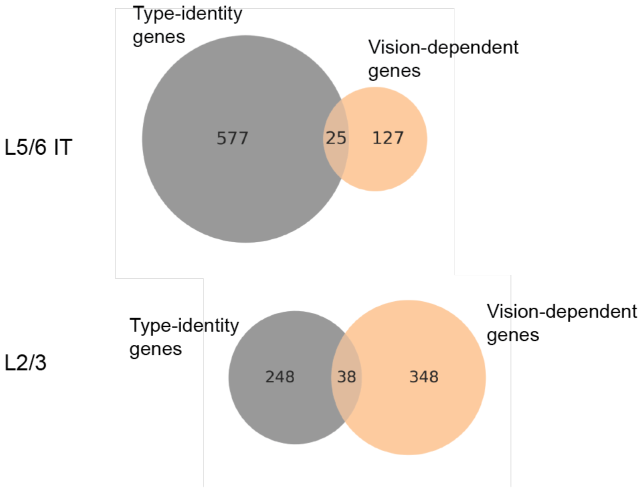

Figure S14. Related to Figure 4. Comparing type-identity gene programs and vision-dependent gene programs in superficial (L2/3) and deep (L5/6) layer intra-telencephalic (IT) neurons.

Venn diagrams showing the numbers and the degree of overlaps between type-identity genes and vision-dependent genes identified in L5/6 IT (upper panel) and L2/3 IT (lower panel) neurons.
