# Supplementary material for: Vision sculpts a continuum of L2/3 cell types in the visual cortex during the critical period
Source: bioRxiv. 2023 Dec 18:2023.12.18.572244. Preprint. [Version 1] doi: 10.1101/2023.12.18.572244 (PMC10769288; doi:10.1101/2023.12.18.572244)
Supplement: Supplementary file 1 [file NIHPP2023.12.18.572244V1-supplement-2.pdf]

bioRxiv preprint doi: https://doi.org/10.1101/2023.12.18.572244; this version posted December 18, 2023. The copyright holder for this preprint (which was not certified by peer review) is the author/funder, who has granted bioRxiv a license to display the preprint in perpetuity. It is made available under aCC-BY 4.0 International license.

Supplemental Figures

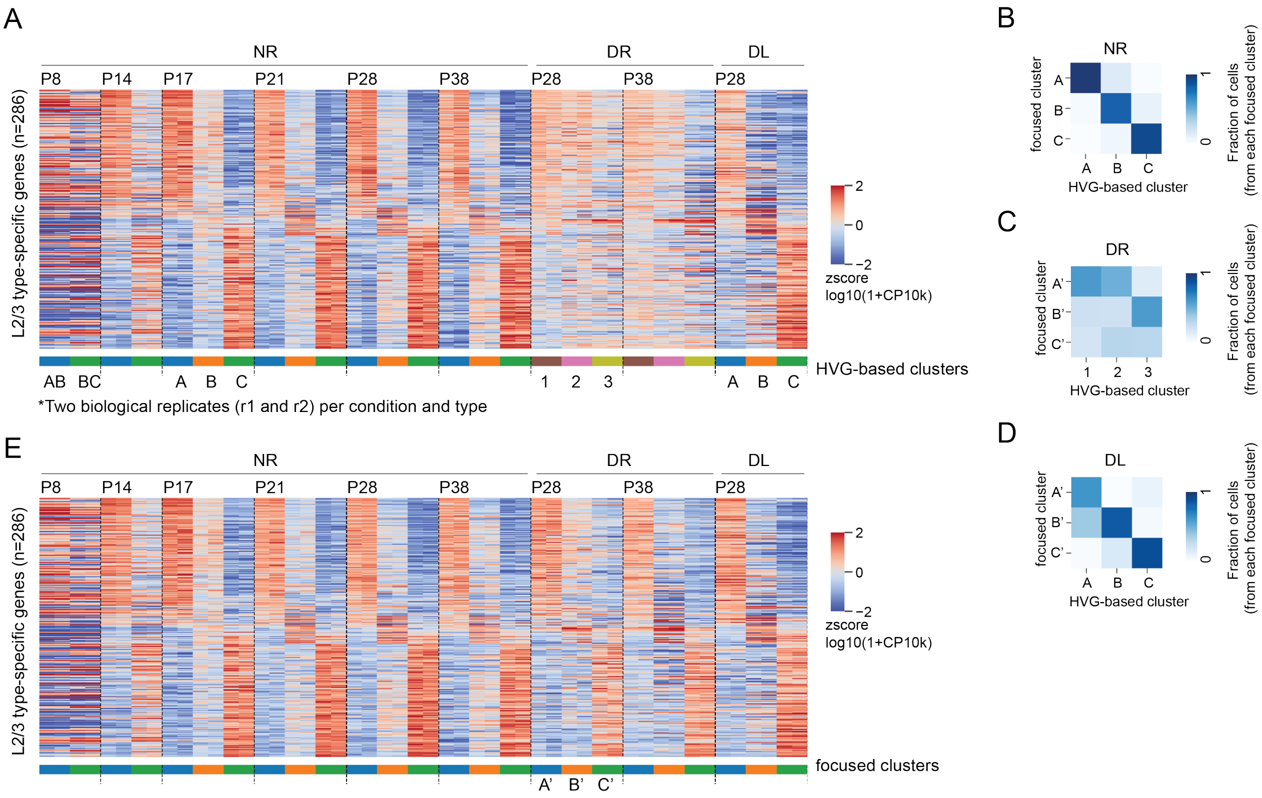

Figure S1. (Related to Figure 1) A focused clustering approach identifies consistent L2/3 types in NR and DR.

A. Expression profiles of L2/3 type-specific genes for HVG-based clusters. **B-D**. Confusion matrix of HVG-based clusters vs focused clusters for NR (**B**), DR (**C**) and DL (**D**). **E.** Same as (A) but for focused clusters.

bioRxiv preprint doi: https://doi.org/10.1101/2023.12.18.572244; this version posted December 18, 2023. The copyright holder for this preprint (which was not certified by peer review) is the author/funder, who has granted bioRxiv a license to display the preprint in perpetuity. It is made available under aCC-BY 4.0 International license.

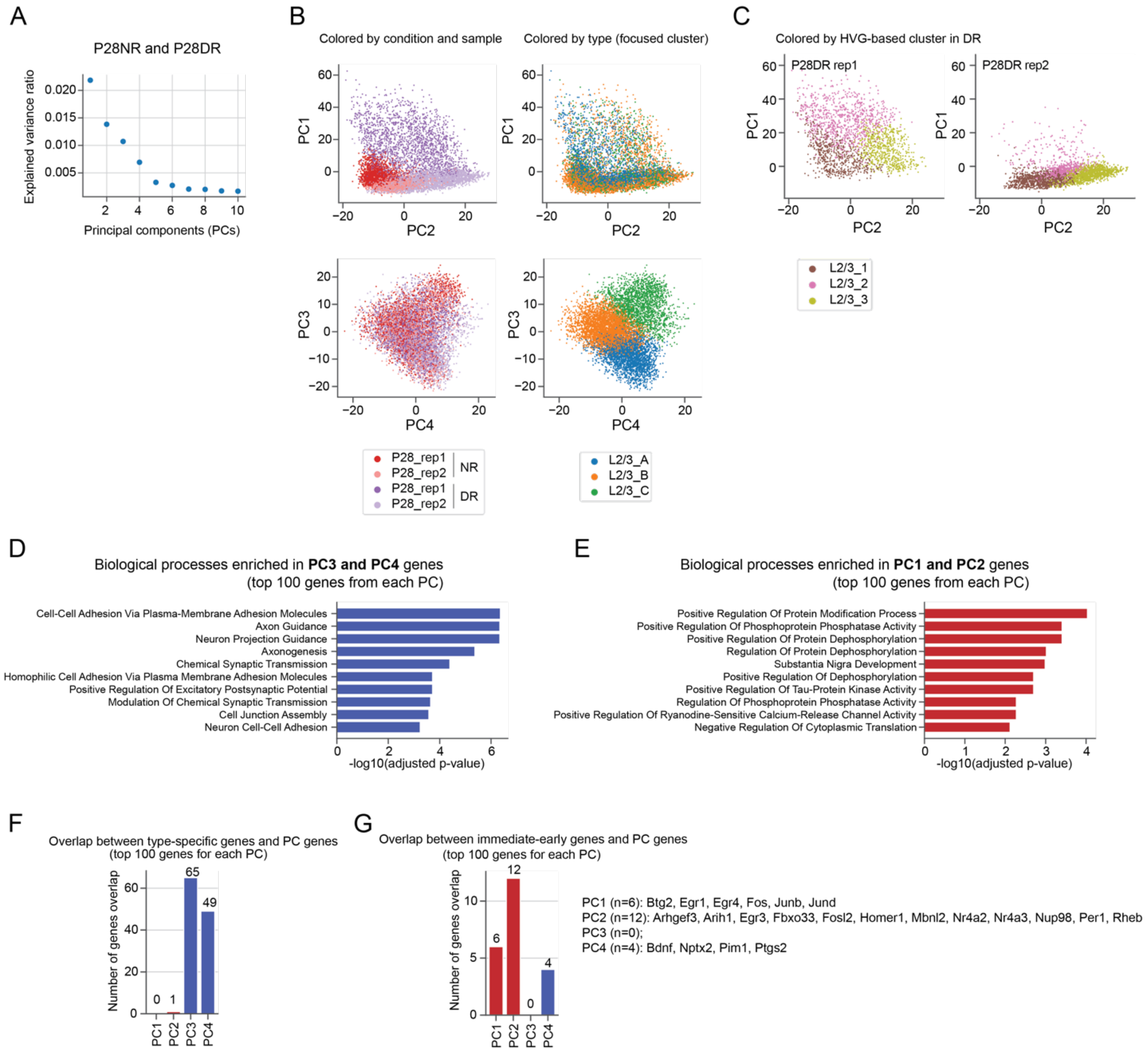

Figure S2. (Related to Figure 1) PCA and Gene Ontology analyses revealed orthogonal components of transcriptomic variations.

A. Fraction of total variance captured within the top 10 principal components (PCs) in P28NR and P28DR L2/3 cells. **B-C.** PC embeddings of P28NR and P28DR cells colored by sample and condition, by focused cluster (B), and by HVG-based cluster for DR cells (C). **D-E.** Gene ontology (GO) analysis showing significantly enriched (adjusted p-value < 0.05) biological processes for PC3/PC4 genes (**D**) and PC1/PC2 genes (**E**), respectively. **F-G.** Barplots showing the number of genes overlapping between PC genes and type-specific genes (**F**), and between PC genes and immediate early genes curated by a previous study[13] (**G**).

bioRxiv preprint doi: https://doi.org/10.1101/2023.12.18.572244; this version posted December 18, 2023. The copyright holder for this preprint (which was not certified by peer review) is the author/funder, who has granted bioRxiv a license to display the preprint in perpetuity. It is made available under aCC-BY 4.0 International license.

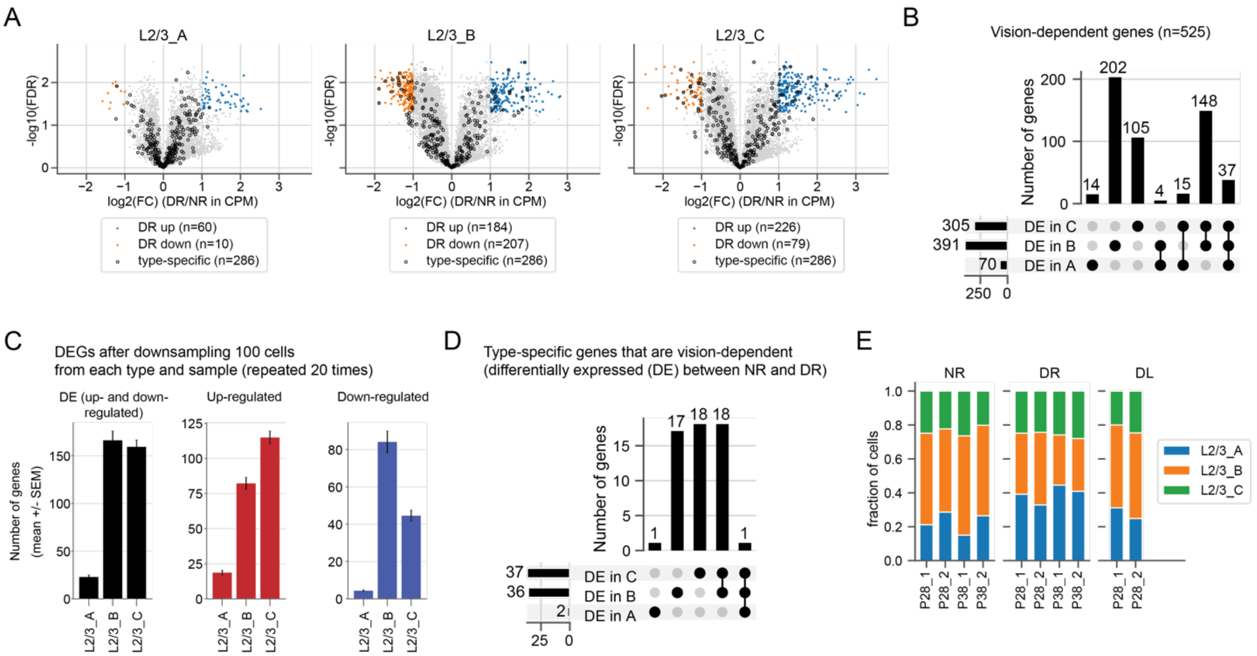

Figure S3. (Related to Figure 2) The differential impact of dark-rearing on L2/3 type-specific genes.

A. Volcano plots highlighting vision-dependent genes for each L2/3 type. **B.** Upset plot showing the overlap between vision-dependent genes across L2/3 types. **C.** Numbers of total vision-dependent genes (left), up-regulated (middle), and down-regulated DEGs (right) in DR after downsampling each L2/3 type to equal number of cells (n=100 per type per sample). Error bar shows standard error of the mean (SEM) derived from 20-time repeated downsampling. **D.** Upset plot showing the overlap between vision-dependent identity genes across L2/3 types. **E.** Relative abundance of type L2/3 A, B, C cells among L2/3 glutamatergic neurons by sample and rearing conditions. NR, normal rearing; DR, dark rearing; DL, dark light.

bioRxiv preprint doi: https://doi.org/10.1101/2023.12.18.572244; this version posted December 18, 2023. The copyright holder for this preprint (which was not certified by peer review) is the author/funder, who has granted bioRxiv a license to display the preprint in perpetuity. It is made available under aCC-BY 4.0 International license.

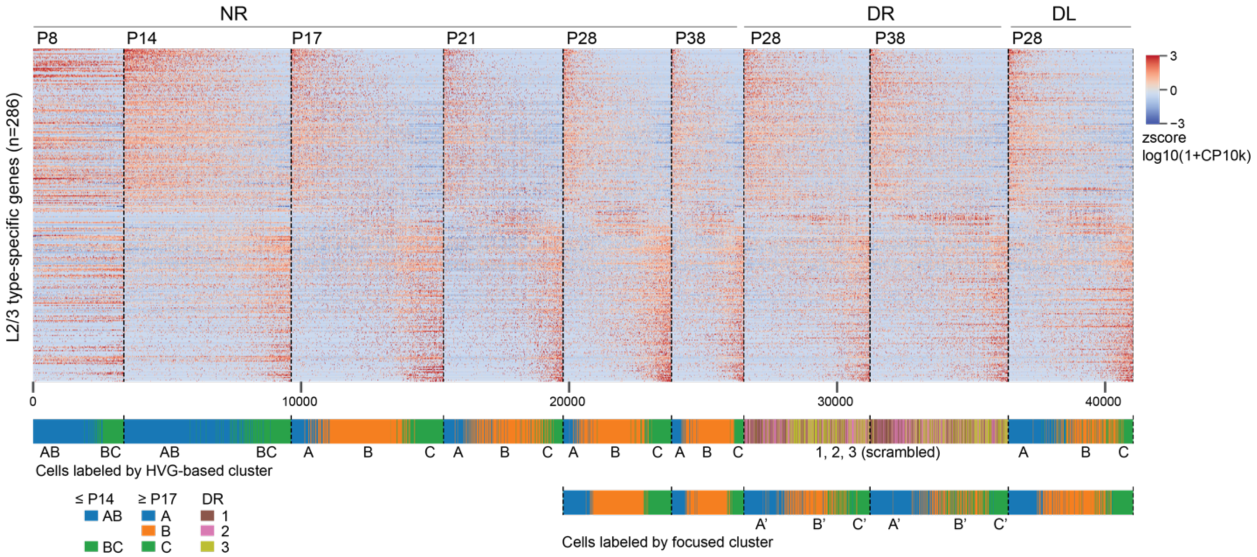

Figure S4. (Related to Figure 3) The L2/3 transcriptomic continuum across time and conditions.

A. Expression profiles of L2/3 type-specific genes for individual cells. Top: Gene expression profiles. Bottom: cells colored by HVG-based and focused clustering as indicated. Cells were ordered by diffusion pseudo-time for each condition (see Methods).

bioRxiv preprint doi: https://doi.org/10.1101/2023.12.18.572244; this version posted December 18, 2023. The copyright holder for this preprint (which was not certified by peer review) is the author/funder, who has granted bioRxiv a license to display the preprint in perpetuity. It is made available under aCC-BY 4.0 International license.

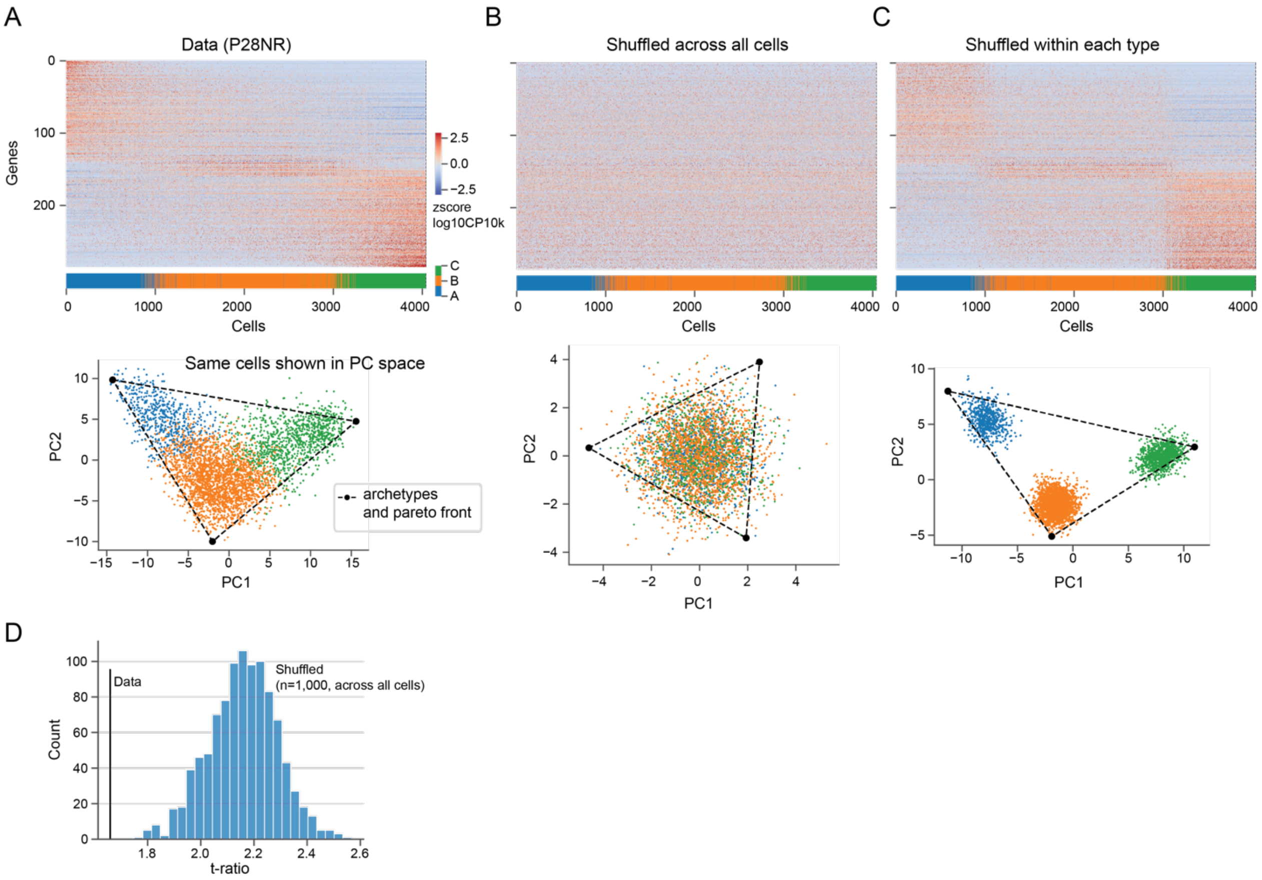

Figure S5. (Related to Figure 3) Data shuffling disrupted the geometry of the L2/3 transcriptomic continuum.

A. Upper panel: expression profiles of L2/3 type-specific genes (n=286) across all L2/3 glutamatergic neurons (n=4,044; P28NR). Expressions were quantified as z-scored, log- and size-normalized counts from scRNA-seq. Cells were ranked by diffusion pseudotime, and colored by type assignment (focused type). Lower panel: PCA embeddings (PC1 and PC2) of L2/3 glutamatergic neurons. Cells were colored by type. The triangular bound represents the Principal Convex Hull. **B.** Same as (A) but after shuffling each gene independently across all cells. **C.** Same as (A) but after shuffling each gene independently across cells within each type. **D.** T-ratios for the data and shuffled data (n=1,000; shuffled across all cells). T-ratio is the ratio between the area of the convex hull and that of the principal convex hull (triangular fit).

bioRxiv preprint doi: https://doi.org/10.1101/2023.12.18.572244; this version posted December 18, 2023. The copyright holder for this preprint (which was not certified by peer review) is the author/funder, who has granted bioRxiv a license to display the preprint in perpetuity. It is made available under aCC-BY 4.0 International license.

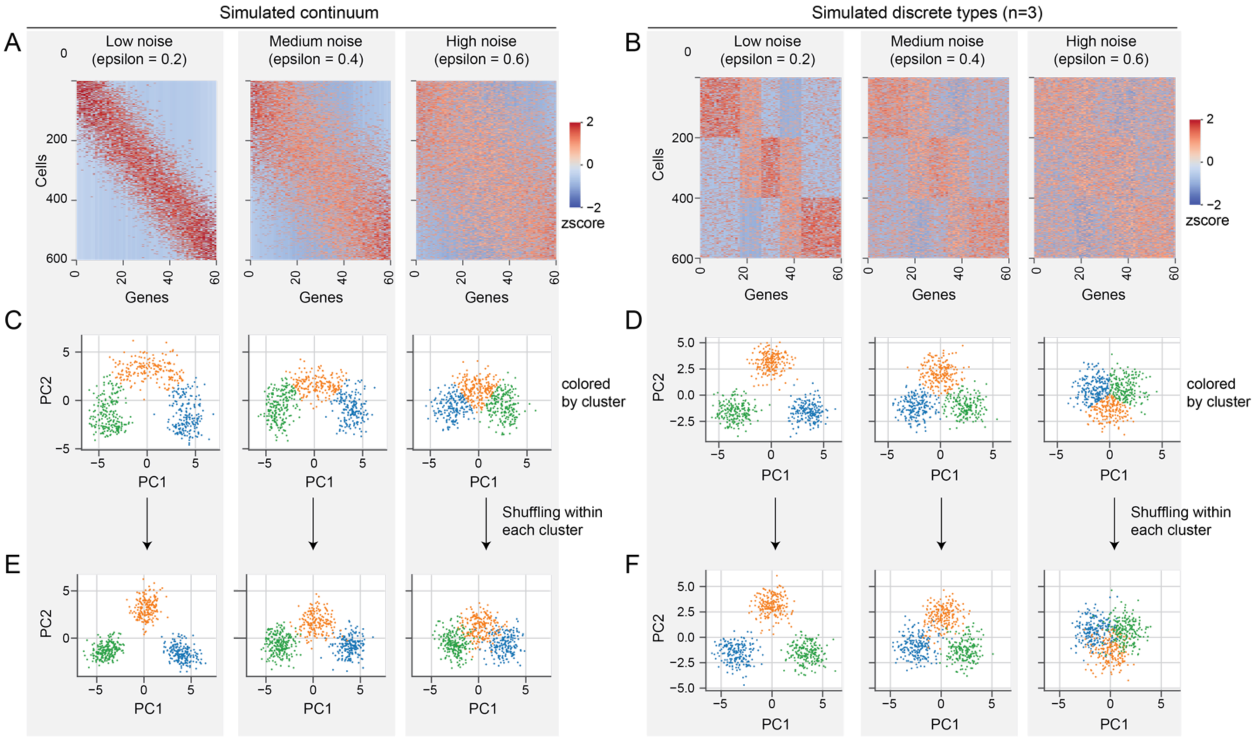

Figure S6. (Related to Figure 3) A data shuffling procedure that split a continuum while preserving discrete types.

A-B. Expression profiles (gene by cell) for simulated continuums (A) and simulated discrete types (B) each with varying degrees of noise level (parameterized by epsilon; see **Methods**). Expression was quantified as z-scores. **C-D.** PCA embeddings (PC1 and PC2) of simulated continuums (C) and simulated discrete types (D). Cells were colored by type. **E-F.** Same as (C-D) but after shuffling each gene independently across all cells within each type. Shuffling genes within a cluster splits a continuum into separate clusters when noise is low (epsilon = 0.2 ~ 0.4). The same procedure has no effect on discrete types whatsoever.

bioRxiv preprint doi: https://doi.org/10.1101/2023.12.18.572244; this version posted December 18, 2023. The copyright holder for this preprint (which was not certified by peer review) is the author/funder, who has granted bioRxiv a license to display the preprint in perpetuity. It is made available under aCC-BY 4.0 International license.

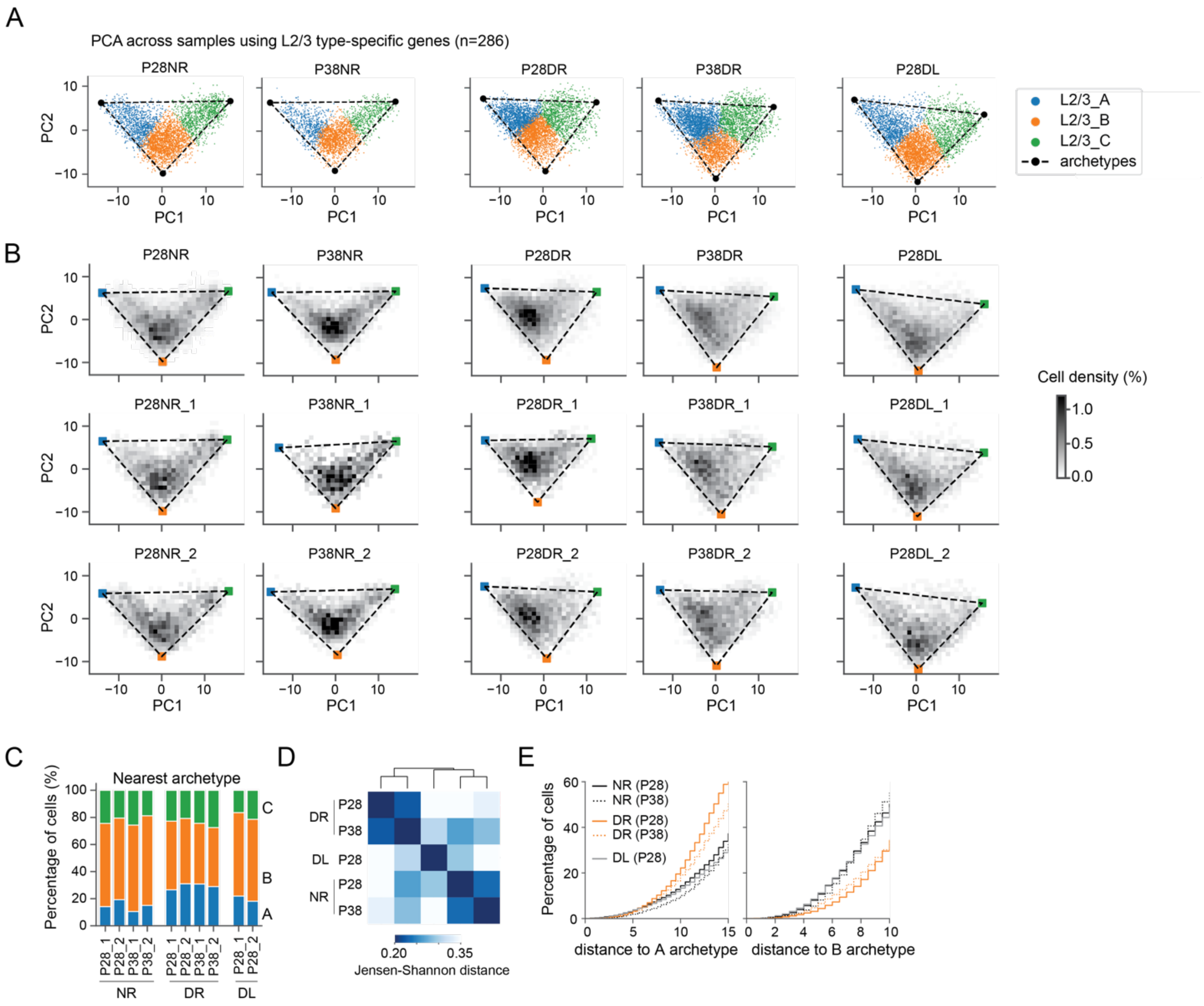

Figure S7. (Related to Figure 3) Dark-rearing shifted cells along the transcriptomic continuum of L2/3 cell types.

A. PCA embeddings (PC1 and PC2 based on type-identity genes) of L2/3 glutamatergic neurons. Cells were colored by type. The vertices of the triangular bound represents archetypes. **B.** Cell density within PC1 and PC2 space for each condition and biological replicate. **C.** Barplot showing the proportion of cells nearest to each archetype for each biological sample. **D.** Pairwise Jensen-Shannon distances between samples of different rearing conditions. **E.** Cumulative percentage of cells as a function of the distance to archetype A (left) and archetype B (right).

bioRxiv preprint doi: https://doi.org/10.1101/2023.12.18.572244; this version posted December 18, 2023. The copyright holder for this preprint (which was not certified by peer review) is the author/funder, who has granted bioRxiv a license to display the preprint in perpetuity. It is made available under aCC-BY 4.0 International license.

Supplemental Tables

Table S1. L2/3 type identity genes (n=286), in which 55 were up- or down-regulated in dark reared mice.
